# Characteristics of gut microbiota in *Penaeus vannamei* shifted with elevated salinity

**DOI:** 10.3389/fmicb.2025.1665547

**Published:** 2025-09-17

**Authors:** Bo Wang, Yang Liu, Xinhua Wu, Yunfei Liu, Ziying Li, Jian Wang, Yingli Lian, Jiayi Tang, Biao Yun, Xiangli Tian

**Affiliations:** ^1^Animal Husbandry and Fisheries Research Center of Guangdong Haid Group Co., Ltd., Guangzhou, China; ^2^State Key Laboratory of Tropical Oceanography, South China Sea Institute of Oceanology, Chinese Academy of Sciences, Guangzhou, China; ^3^Key Laboratory of Microecological Resources and Utilization in Breeding Industry, Ministry of Agriculture and Rural Affairs, Guangdong Haid Group Co., Ltd., Guangzhou, China; ^4^The Key Laboratory of Mariculture, Ocean University of China, Ministry of Education, Qingdao, China; ^5^Bohai Seafoods Co., Ltd., Binzhou, China

**Keywords:** co-occurrence network, community assembly, gut bacterial community, salinity, *Penaeus vannamei*

## Abstract

An increasing number of studies have evaluated the effects of host, dietary, and environmental factors on the gut microbial community of *Penaeus vannamei*. However, the characteristics of the gut microbial community of this species in hypersaline aquaculture environments have not yet been clarified. Our findings demonstrate that salinity has a strong impact on the gut bacterial community of shrimp. The alpha diversity of the gut bacterial community of shrimp decreased with salinity. Significant differences in the composition and abundance of the core gut bacterial taxa were observed among ponds with varying salinity, and only 13 shared core operational taxonomic units (OTUs) were identified; the abundance of potential opportunistic pathogens decreased significantly in hypersaline environments. Salinity is identified as a critically important environmental factor affecting the structure of the gut bacterial community of shrimp in hypersaline environments. The structure of the gut bacterial community of shrimp was distinct at salinities of 31–39 and 47–55, and the predicted functions differed at salinities of 31–47 and 55 based on 16S rRNA gene prediction using PICRUSt2 and principal coordinate analysis. Network analysis showed that higher salinity was associated with less connectivity and cooperation among species. Neutral Community Model analysis and the normalized stochasticity ratio revealed that stochastic processes were dominant at lower salinity; however, deterministic processes became more important as salinity increased. In addition, the community-level habitat niche breadths of the gut bacterial community decreased with salinity, which further confirmed this trend. These findings provide new insights into the characteristics of the gut bacterial community of shrimp in hypersaline environments and would contribute to the improvement of farming health management of shrimp in hypersaline ponds aquaculture practices.

## 1 Introduction

Gut microbiota are critical for maintaining host health by regulating growth performance, nutrient absorption, metabolic processes, immune responses, and the maintenance of homeostasis (Hooper et al., 2002; Hooper and Macpherson, 2010; Boulangé et al., 2016; Cornejo-Granados et al., 2017; Cani et al., 2019). Studies of the microbial characteristics of the host gut and the factors affecting their characteristics are thus critically important. Previous studies have revealed a large number of diseases associated with dysbiosis of the host intestinal microbiota (Dai et al., 2020; de Souza Valente et al., 2020; Huang et al., 2020; Zhang et al., 2021). Many studies have reported that environmental factors, such as the salinity, ammonia nitrogen, temperature, and pH of culture water, have significant effects on the intestinal microbiota of aquatic animals (Sullam et al., 2012; Huang et al., 2018; Hou et al., 2020; Wang et al., 2021). In recent years, the differential effects of seawater and freshwater on the intestinal microbiota of aquatic animals have been investigated; however, the effects of high salinity on the intestinal microbiota of aquatic animals have not been widely examined.

The Pacific white shrimp (*Penaeus vannamei*), a native species inhabiting the eastern Pacific coast from Mexico to Peru (FAO, 2018), can grow and survive at a wide salinity range from 0.5 to 60. This species is cultivated throughout the world for its rapid growth rate, tender flesh, and high nutritional value (Jin et al., 2018). China is currently the world’s largest white shrimp producer. The Pacific white shrimp seawater cultivation area was 1.63 × 10^5^ ha in 2022, and 1.34 million tons of Pacific white shrimp were produced in this same year (China Fishery Statistical Yearbook, 2023). *P. vannamei* can adapt to a wide range of environmental salinities; thus, clarifying the responses of *P. vannamei* to various salinities is critically important for ensuring the efficacy of aquaculture management. Understanding the effects of salinity on the gut microbial community might shed light on the mechanisms underlying differences in the growth performance of *P. vannamei* at different salinities. Most previous studies have focused on characterizing differences in the gut bacterial community of shrimp cultured in freshwater and seawater (Roy et al., 2010; Hou et al., 2020); however, few studies have determined the characteristics of the gut bacterial community of shrimp cultured in hypersaline environments (salinity more than 35).

In northern China, especially in Liaoning, Tianjin, Shandong, and Hebei Provinces, which comprise the coasts along the Yellow and Bohai Seas, there is a vast area of primary salt evaporation ponds for salterns, which cover an area greater than 1.33 × 10^5^ ha (unpublished data), and shrimp aquaculture in this area has greatly increased the economic benefits of salterns. In these types of ponds, the farming area is large (these are commonly referred to as “Dawangzi” in Chinese, which means large ponds in salt pan), and the salinity is high; the stocking density and survival rate of shrimp are low in these areas, but the flavor of shrimp is improved. Few studies have examined the ecological characteristics of shrimp and environments in these aquaculture ecosystems. There is thus a need for more studies of the gut microbiota of *P. vannamei* cultured in hypersaline environments. Our study was conducted in Binzhou, Shandong Province, which has a large number of primary salt evaporation ponds that cover an area of more than 5.33 × 10^4^ ha, and the salinity ranges from 31 to 65. The average production of shrimp per ha in such ponds is lower than that in conventional culture ponds (Supplementary Table S1); there is thus much room for improvement in the natural resource utilization and economic benefits of salterns.

Here, we investigated the primary salt evaporation ponds for shrimp culture in Bohai Seafoods Co., Ltd., (Binzhou, China) to characterize the responses of the gut bacterial community of *P. vannamei* to various hypersaline environments. The findings from the present study would provide new insights into the characteristics of the gut bacterial community of shrimp in hypersaline environments and contribute to the improvement of farming health management in hypersaline ponds.

## 2 Materials and methods

### 2.1 Sample collection and physicochemical analysis

A total of 120 shrimp were collected from four culture ponds [six sampling positions per pond, five shrimp per position were collected from Bohai Seafoods Co., Ltd., (Binzhou, China)] (38.03°–38.06°N, 117.56°–118.01°E), in September 2020, and the 5 shrimp per replicate pooled into a single sample. The shrimp in the four ponds came from the same batch of postlarvae and their rearing days (~100 days) were consistent. Four shrimp culture ponds were randomly sampled, A (106.7 ha in area), B (112.7 ha in area), C (133 ha in area), and D (167 ha in area); the depth of the water in these ponds ranged between 0.5 and 3 m, and the salinity was 31 ± 0.85, 39 ± 1.23, 47 ± 0.62, and 55 ± 1.74, respectively. Intestinal samples were aseptically dissected from each shrimp using sterile surgical instruments and immediately transferred to individual 2-mL sterile centrifuge tubes containing. All samples were flash-frozen in liquid nitrogen and stored at −80 °C until genomic DNA extraction. The average body weight of shrimp ranged from 14 to 15 g. For each pond, six water samples (1.0 L water of each sample) were collected at depths of 0.5 m below the surface using sterile bottles, and samples were immediately placed on ice before being filtered through 0.45 μm glass fiber filter membranes using peristaltic pumps; the filtered water was immediately stored at 4 °C and then transported to the laboratory for water quality analysis. Temperature, pH, DO, and salinity were measured on-site using a YSI handheld multi-parameter instrument (Model YSI ProPlus, YSI Incorporated, United States). Total nitrogen (TN), total phosphorus (TP), ammonia nitrogen (NH_4_^+^-N), nitrate nitrogen (NO_3_^−^-N), nitrite nitrogen (NO_2_^−^-N), and sulfide and active phosphorus (PO_4_^3−^-P) were measured using an automatic discrete analyzer (Model CleverChem 380, DeChem-Tech, Germany). The complete water quality analysis results for all four ponds are provided in the Supplementary Table S2.

### 2.2 DNA extraction, amplification, and sequencing

Genomic DNA from shrimp guts was extracted using a PowerSoil DNA Isolation Kit (Mobio, Carlsbad, CA, United States) according to the manufacturer’s protocol. The V4 region of the 16S rRNA gene from bacteria and archaea was amplified using the primers 515FmodF (5′-GTGYCAGCMGCCGCGGTAA-3′) and 806RmodR (5′-GGACTACNVGGGTWTCTAAT-3′). PCR reactions were performed in 20 μL mixtures containing 4 μL of 5 × FastPfu Buffer, 2 μL of 2.5 mM dNTPs, 0.8 μL of each primer (5 μM), 0.4 μL of FastPfu polymerase, and 10 ng of purified DNA as a template. The thermal cycling parameters were as follows: 95 °C for 3 min; 25 cycles of 95 °C for 30 s, 55 °C for 30 s, and 72 °C for 45 s; and a final extension at 72 °C for 10 min. The quality of the PCR products was detected using 1.5% agarose gel electrophoresis. Subsequently, the purified PCR products were sequenced on an Illumina MiSeq platform by Majorbio Bio-pharm Technology Co., Ltd. (Shanghai, China). Raw 16S rRNA sequence data were submitted to the NCBI Sequence Read Archive (SRA) database under accession number PRJNA833737.

### 2.3 Sequence data processing

Raw sequencing data generated from the Illumina MiSeq platform were merged with FLASH (version 1.2.11) (Mago and Salzberg, 2011). For quality control, merged sequences were processed using the Quantitative Insights into Microbial Ecology software (QIIME version 1.9.1) (Caporaso et al., 2010). All chimeric and normal-quality reads were removed using the UCHIME algorithm (Edgar et al., 2011), and the qualified sequences were clustered into operational taxonomic units (OTUs) using a 97% similarity threshold via UPARSE (version 7.0.1090) (Edgar, 2013). The most abundant sequence of each OTU was used as the representative sequence; taxonomic annotations of the sequences were obtained using the RDP Classifier algorithm (Wang et al., 2007) and the SILVA database 138, and a close relative could be identified for each OTU. Rarefaction curves based on the observed features and Shannon indexes were drawn to determine the sequencing depth. QIIME software (version 1.9.1) was used to analyze alpha diversity, including community richness indexes (ACE and Chao1), community diversity indexes (Shannon and Simpson), and Good’s coverage index. The Shannon and Simpson indexes are commonly used to quantify diversity, and the Chao1 and ACE indexes are used to quantify richness. Principal coordinate analysis (PCoA) and PERMANOVA analysis were conducted to evaluate differences in bacterial community structure based on Bray-Curtis distance metrics in R software, and QIIME was used to construct phylogenetic trees based on Bray-Curtis distances to investigate the relationships among samples. Linear discriminant analysis (LDA) effect size (LEfSe) was used to determine statistically differential taxa (biomarkers) (Segata et al., 2011). To investigate the relationships between microbial community structure and environmental factors, redundancy analysis (RDA) was performed using CANOCO 5.0 software. Analysis of variance (ANOVA) followed by stepwise ordination was used to determine the significance of the overall model and perform RDA; the significance of each of the environmental variables was then determined based on the *p*-values. The concept of the core microbiome considers persistent and highly abundant microbes in a microbial community, which is a stable community (Shade and Handelsman, 2012). The core gut microbiome of shrimp comprised the microbes that were present at all regional sites and in ≥80% of all samples with average relative abundances ≥0.1% (Wu et al., 2019).

### 2.4 Co-occurrence pattern analysis

Co-occurrence network analysis was performed on the 100 most abundant bacterial genera. The CoNet plug-in of Cytoscape 3.9.0 was used to construct the network (Faust et al., 2012). The network was constructed using four different algorithms: Pearson correlation, Spearman correlation, Kullback–Leibler distance, and Bray-Curtis distance. The use of multiple algorithms effectively reduces the likelihood of constructing erroneous network relationships, which yields more realistic and accurate network relationships. The final network was obtained through several steps such as Permutations, Bootstrapping, and Restoring using Brown’s *p*-value merging method and Benjamini-Hochberg’s multiple test correction method. The topological properties of the network were analyzed by Network Analyzer in Cytoscape 3.9.0.

### 2.5 Prediction of gut bacterial community functions

The OTU table was normalized by dividing their abundances by their known or predicted 16S rRNA gene copy number abundances prior to making final metagenomic predictions. Bacterial community functions were predicted from 16S rRNA sequencing data using PICRUSt2, and the predicted functions were annotated at levels 1, 2, and 3 using the Kyoto Encyclopedia of Genes and Genomes (KEGG) database (Langille et al., 2013). A principal coordinate analysis (PCoA) was performed to analyze the similarity in the content of level 3 predicted functions for the gut bacterial community based on the Bray-Curtis distance; PERMANOVA was used to evaluate the significance of differences between groups. One-way ANOVA was performed to evaluate the significance of differences in level 2 and level 3 predicted functions.

### 2.6 Bacterial community assembly analysis

A Neutral Community Model (NCM) was used to determine the potential contribution of stochastic processes to gut bacterial community assembly by predicting the relationship between the occurrence frequency of OTUs and their relative abundance (Sloan et al., 2010). The *R*^2^ value represents the goodness of fit of the model, which ranges between 0 to 1. The higher R^2^ indicates that the community assembly is closer to stochastic process. NCM was performed in R version 4.2.2 using the “Hmisc,” “minpack.lm,” and “stats4” packages. We also calculated the normalized stochasticity ratio (NST) to quantify the relative importance of stochastic and deterministic processes in community assembly using 50% as the threshold for inferring whether assembly was more deterministic (<50%) or stochastic (>50%) (Ning et al., 2019). This analysis was performed in R version 4.2.2 using the “NST” package. Habitat niche breadth was calculated using Levin’s niche breadth index, which was determined as follows:


$$B_{j}=1/\sum_{i=1}^{N}P_{\mathrm{ij}}^{2}$$

where *B*_j_ represents the habitat niche breadth of OTU_j_ in a metacommunity, *N* represents the total number of communities of each metacommunity, and *P*_ij_ represents the proportion of OTU *j* in community *I* (Pandit et al., 2009; Wu et al., 2017). A high *B*-value indicates that the OTU is widespread and uniform at a variety of sites, which indicates a wide habitat niche breadth. We calculated the average *B* values of all taxa in a single community (Bcom) as an indicator of habitat niche breadth at the community level. The analysis was performed in R version 4.2.2 using the “niche.width” function and “spaa” package (Zhang, 2013; Zhang M. et al., 2016).

### 2.7 Statistical analysis

Results were expressed as mean ± standard error of the mean. One-way ANOVA followed by Duncan’s multiple-range test was used to compare the significance of differences in alpha diversity indexes among gut samples using SPSS22.0. A value of *p* < 0.05 was considered statistically significant.

## 3 Results

### 3.1 16S rRNA gene sequencing and analysis of bacterial diversity in shrimp gut

A total of 1,232,430 high-quality sequences with an average of 61,296 sequences (35,873 to 148,098) were obtained (Supplementary Table S3); after subsampling 35,873 minimum sequences per sample, 717,460 sequences were retained. Based on analysis of the rarefaction curve and Shannon-Wiener curve, the sequencing depth was sufficient for sampling all bacterial diversity (Supplementary Figure S1). Sequences with 97% similarity were clustered into a class, and a total of 23,167 OTUs were identified. The numbers of OTUs detected in each sample ranged from 350 to 1,476 (Supplementary Table S3).

**Figure 1:**
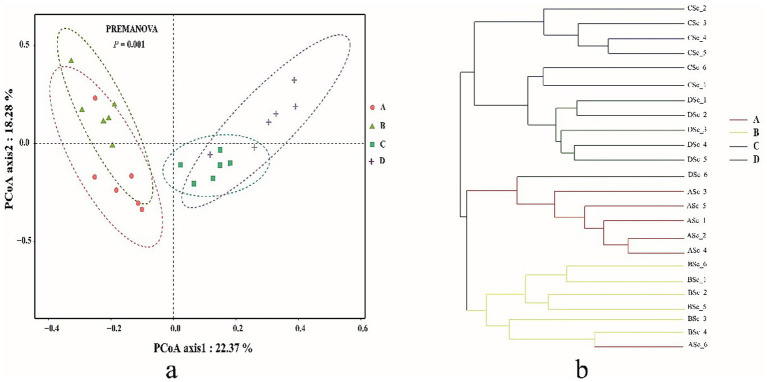
The principal co-ordinates analysis (PCoA) **(a)** and the hierarchical clustering tree **(b)** of gut bacterial community in shrimp. A, B, C, D mean salinity 31 ± 0.85, 39 ± 1.23, 47 ± 0.62, and 55 ± 1.74.

Bacterial community complexity was estimated and compared by calculating the diversity index and richness index for all samples. The diversity (Shannon and Simpson) of the shrimp gut bacterial community at salinities A (31 ± 0.85) and B (39 ± 1.23) was significantly higher than that at salinities C (47 ± 0.62) and D (55 ± 1.74) (*p* < 0.05) (Table 1). The richness (Chao1 and ACE) of the gut bacterial community of shrimp at salinities A and B was also significantly higher than that at salinities C and D (*p* < 0.05). Although the differences were not always statistically significant, the richness and diversity of gut bacterial community of shrimp were highest at salinity A, followed by salinity B, C, and D. The Good’s coverage index for each sample was greater than 99.30% (99.308 to 99.679%), suggesting that the sequencing depth was sufficient for microbial community analysis. A PCoA, based on the Bray-Curtis distance algorithm at the OTU level, was performed to characterize the beta diversity of the gut bacterial community of shrimp. Significant separation was observed between the structure of the gut bacterial community at salinities A and B and that at salinities C and D along the PC1 axis, (*p* < 0.05) (Figure 1). The PC1 and PC2 axes explained 40.65% of the variation between samples (Figure 1a). To investigate whether there are significant differences in the bacterial community between samples in specific evolutionary lineages, a hierarchical cluster tree based on the Bray-Curtis distance algorithm at the OTU level was constructed (Figure 1b). The gut bacterial community samples at salinities A and B were clustered on the same branch, suggesting that the gut bacterial samples at salinities A and B were closely related; the gut bacterial community samples at salinities C and D were clustered on the same branch with the exception of the DSc_6, suggesting that the gut bacteria samples from salinities C and D were closely related.

**Table 1:** Richness and diversity indexes for gut bacterial community in shrimp at different salinity.

Sample	Shannon	Simpson	Chao1	ACE	Goods’ coverage
A	3.98 ± 0.38^b^	0.09 ± 0.01^a^	1977.59 ± 104.85^b^	2737.66 ± 73.30^c^	0.99 ± 0.00^a^
B	3.80 ± 0.05^b^	0.13 ± 0.03^a^	1814.31 ± 165.04^b^	2550.98 ± 94.89^c^	0.99 ± 0.00^a^
C	3.32 ± 0.02^a^	0.15 ± 0.02^ab^	1208.75 ± 36.41^a^	1526.48 ± 139.67^b^	0.99 ± 0.00^a^
D	3.06 ± 0.19^a^	0.17 ± 0.01^b^	1090.89 ± 11.68^a^	1104.39 ± 17.15^a^	0.99 ± 0.00^a^

Data were shown as mean ± S. E. (*n* = 5). Data in the same column sharing the different superscript letter are significantly different as determined by one-way ANOVA test (*p* < 0.05). A, B, C, D mean salinity 31 ± 0.85, 39 ± 1.23, 47 ± 0.62, and 55 ± 1.74.

### 3.2 Taxonomic composition of the gut bacterial community and biomarker analysis

**Figure 2:**
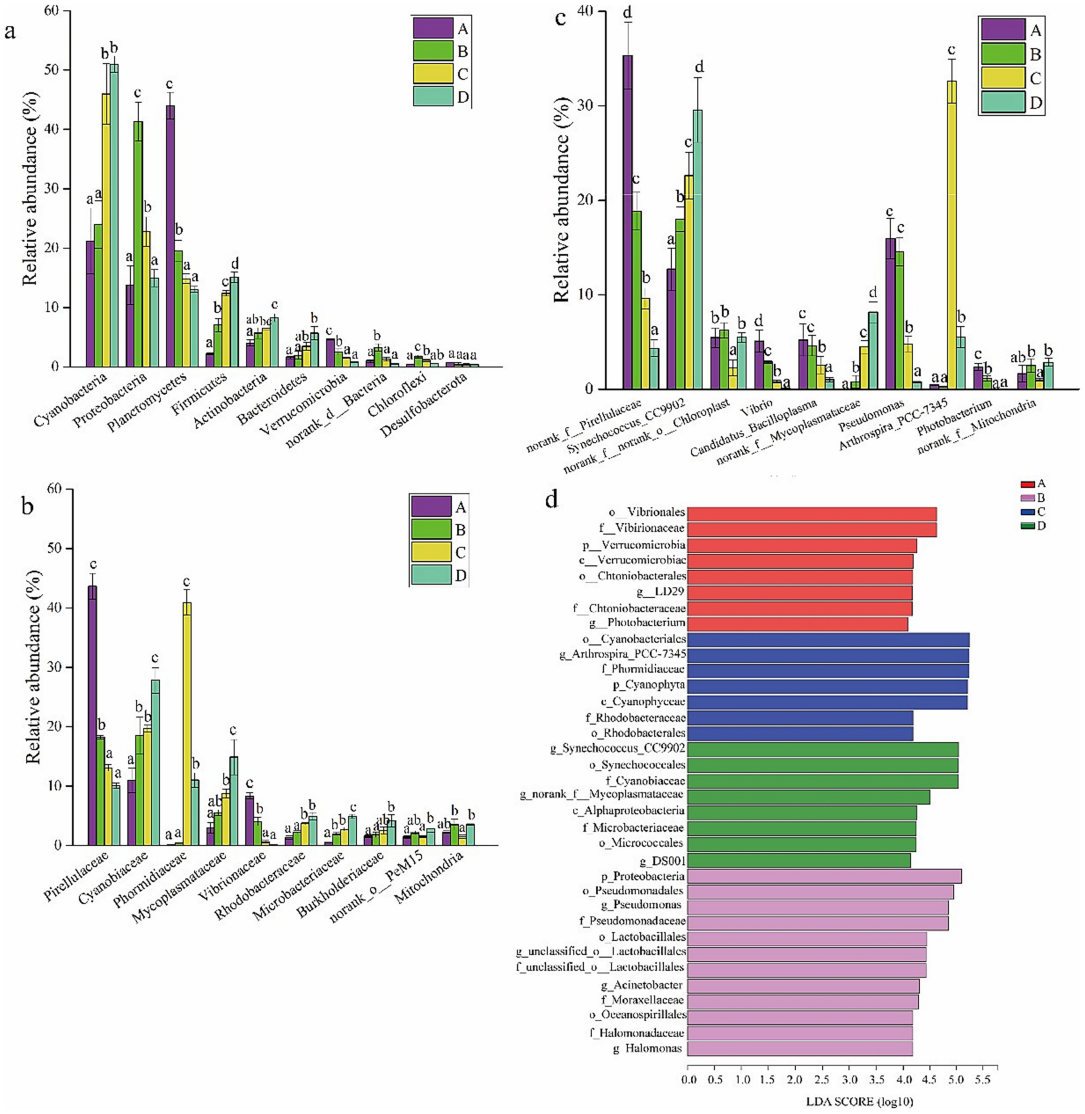
Microbial composition of gut bacterial taxa at phylum level **(a)**, at family level **(b)**, at genus level **(c)**. Data in the same column sharing the different superscript letter are significantly different as determined by one-way ANOVA test (*p* < 0.05). The linear discriminant analysis (LDA) effect size (LEFSe) identified the differentially abundant taxa in four ponds **(d)**. Only taxa meeting an LDA significant threshold of > 4.0 are shown. A, B, C, D mean salinity 31 ± 0.85, 39 ± 1.23, 47 ± 0.62, and 55 ± 1.74.

To compare the gut bacterial community composition of shrimp at the four salinities, the bacterial profiles at the phylum, family, and genus levels were evaluated. Cyanobacteria, Proteobacteria, Planctomycetes, Firmicutes, and Actinobacteria were the five predominant phyla (Figure 2a). The relative abundance of Cyanobacteria, Firmicutes, Actinobacteria, and Bacteroidetes increased with salinity (Figure 2a). The relative abundance of Proteobacteria, norank_d_Bacteria, and Chloroflexi first increased and then decreased with salinity (Figure 2a). The relative abundance of Planctomycetes and Verrucomicrobia decreased with salinity (Figure 2a). Pirellulaceae, Cyanobiaceae, Phormidiaceae, and Mycoplasmataceae were the most abundant families (Figure 2b). The relative abundance of Cyanobiaceae, Mycoplasmataceae, Rhodobacteraceae, Microbacteriaceae, and Burkholderiaceae increased with salinity, and they were significantly enriched in the gut of shrimp at salinities C and D (*p* < 0.05) (Figure 2b). The relative abundance of some potential opportunistic pathogens such as *Vibrio*, *Pseudomonas*, *Candidatus_Bacilloplasma*, and *Photobacterium* was significantly higher at salinities A and B than at salinities C and D (Figure 2c). *Synechococcus_CC9902*, *norank_f__Mycoplasmataceae*, and *Arthrospira_PCC-7345* were most abundant at salinities C and D (Figure 2c).

To identify the classified bacterial taxa showing significant differences in abundance among gut samples at the four salinities, a biomarker analysis using the linear discriminant analysis (LDA) effect size (LEFSE) method was performed. Statistically significant differences were observed for 35 bacterial taxa among the four salinities at an LDA threshold of 4.0 (Figure 2d). Eight taxa were highly abundant in the shrimp gut samples at salinity A, including Vibrionales (order), Vibrionaceae (family), Verrucomicrobia (phylum), Verrucomicrobiae (class), Chthoniobacterales (order), *Sphingobium yanoikuyae* (genus), Chthoniobacteraceae (family), and *Photobacterium* (genus). Twelve taxa were highly abundant in the shrimp gut samples at salinity B, including Proteobacteria (phylum), Pseudomonadales (order), *Pseudomonas* (genus), Pseudomonadaceae (family), Lactobacillales (order), *unclassified_o__Lactobacillales* (genus), unclassified_o__Lactobacillales (family), *Acinetobacter* (genus), Moraxellaceae (family), Oceanospirillales (order), Halomonadaceae (family), and *Halomonas* (genus). Seven taxa were highly abundant in shrimp gut samples at salinity C, including Cyanobacteriales (order), *Arthrospira_PCC-7345* (genus), Phormidiaceae (family), Cyanophyta (phylum), Cyanophyceae (class), Rhodobacteraceae (family), and Rhodobacterales (order). Eight taxa were highly abundant in shrimp gut samples at salinity D, including *Synechococcus_CC9902* (genus), Synechococcales (order), Cyanobiaceae (family), *norank_f__Mycoplasmataceae* (genus), Alphaproteobacteria (class), Microbacteriaceae (family), Micrococcales (order), and *DS001* (genus). Some characters at higher taxonomic levels are unlikely to be effective biomarkers because they include various genera and species. Therefore, characters at the genus level can potentially be more effective biomarkers. *Photobacterium* (genus) and *Sphingobium yanoikuyae* (genus) can serve as biomarkers at salinity A (31 ± 0.85); *Pseudomonas* (genus), *unclassified_o__Lactobacillales* (genus), *Acinetobacter* (genus), and *Halomonas* (genus) can serve as biomarkers at salinity B; *Arthrospira_PCC-7345* (genus) can serve as a biomarker at salinity C; and *Synechococcus_CC9902* (genus), *norank_f__Mycoplasmataceae* (genus), and *DS001* (genus) can serve as biomarkers at salinity D.

### 3.3 Core bacterial taxa and environmental drivers of gut bacterial community

**Figure 3:**
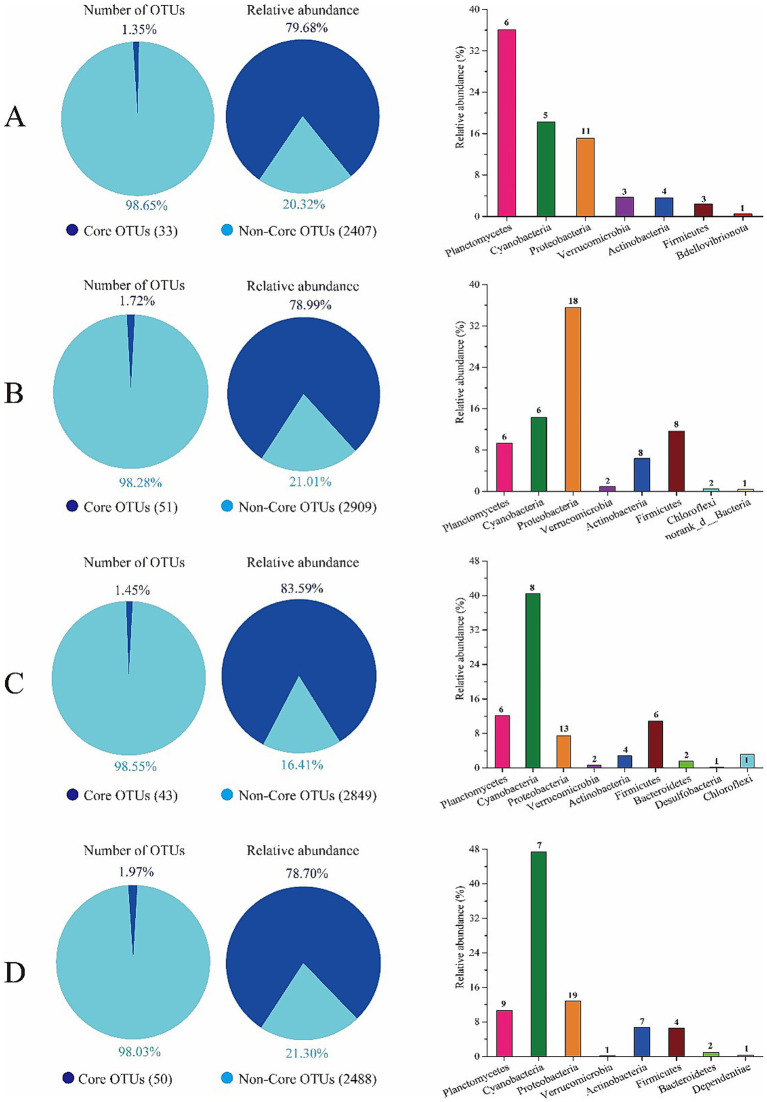
Composition and abundance of the core OTUs in gut bacterial community of shrimp. **(A)** Thirty-three OTUs were identified as the core OTUs in salinity A, accounting for 79.68% of all gut bacterial sequences obtained, which were affiliated to Planctomycetes, Cyanobacteria, Proteobacteria, Verrucomicrobia, Actinobacteria, Firmicutes, and Bdellovibrionota. **(B)** Fifty-one OTUs were identified as the core OTUs in salinity B, accounting for 78.99% of all intestine bacterial sequences obtained, which were affiliated to Planctomycetes, Cyanobacteria, Proteobacteria, Verrucomicrobia, Actinobacteria, Firmicutes, Chloroflexi, and norank_d_Bacteria. **(C)** Forty-three OTUs were identified as the core OTUs in salinity C, accounting for 83.59% of all intestine bacterial sequences obtained, which were affiliated to Planctomycetes, Cyanobacteria, Proteobacteria, Verrucomicrobia, Actinobacteria, Firmicutes, Bacteroidetes, Desulfobacteria, and Chloroflexi. **(D)** Fifty OTUs were identified as the core OTUs in salinity D, accounting for 78.70% of all intestine bacterial sequences obtained, which were affiliated to Planctomycetes, Cyanobacteria, Proteobacteria, Verrucomicrobia, Actinobacteria, Firmicutes, Bacteroidetes, and Dependentiae.

The core taxa of the gut bacterial community of shrimp at the four salinities were identified based on the frequency and abundance of OTUs. Approximately 1.35, 1.72, 1.45, and 1.97% of OTUs comprised core OTUs at salinities A, B, C, and D, which accounted for 79.68, 78.99, 83.59, and 78.70% of all obtained gut bacterial sequences, respectively (Figure 3; Supplementary Table S5). The core gut bacterial OTUs of shrimp at salinity A were in the phyla Planctomycetes, Cyanobacteria, Proteobacteria, Verrucomicrobia, Actinobacteria, Firmicutes, and Bdellovibrionota. The core gut bacterial OTUs of shrimp at salinity B were in the phyla Planctomycetes, Cyanobacteria, Proteobacteria, Verrucomicrobia, Actinobacteria, Firmicutes, Chloroflexi, and norank_d_Bacteria. The core gut bacterial OTUs of shrimp at salinity C were in the phyla Planctomycetes, Cyanobacteria, Proteobacteria, Verrucomicrobia, Actinobacteria, Firmicutes, Bacteroidetes, Desulfobacteria, and Chloroflexi. The core gut core bacterial OTUs of shrimp at salinity D were in the phyla Planctomycetes, Cyanobacteria, Proteobacteria, Verrucomicrobia, Actinobacteria, Firmicutes, Bacteroidetes, and Dependentiae. There were 13 shared core taxa among the core bacterial community of shrimp at salinities A, B, C, and D (Supplementary Figure S2). The 13 shared core OTUs were affiliated with *Rhodococcus*, Pseudomonas, *Mycobacterium*, *uncultured Planctomycetaceae bacterium*, *uncultured Actinomycetales bacterium*, *Delftia tsuruhatensis*, *Planctomycetaceae bacterium D2*, *Blastopirellula*, *Synechococcus_CC9902*, *uncultured Pirellulaceae bacterium*, *Ralstonia*, *Sphingobium yanoikuyae*, and *Microbacteriaceae bacterium CL-Dokdo102* (Supplementary Table S4). In addition, *Vibrio* OTU1121 (4.98%), *Photobacterium* OTU1254 (2.34%), *Candidatus Bacilloplasma* OTU18492 (0.75%), *Vibrio* OTU267 (0.58%), *Mycobacterium* OTU16545 (0.4%), *Ralstonia* OTU1370 (2.69%), and *Pseudomonas* OTU47 (0.23%) were identified as core OTUs at salinity A; *Pseudomonas* OTU18484 (16.94%), *Candidatus Bacilloplasma* OTU75 (4.43%), *Photobacterium* (1.11%), *Candidatus Bacilloplasma* OTU18492 (0.74%), *Vibrio* OTU1121 (0.65%), *Pseudomonas* OTU24521 (0.27%), *Vibrio* OTU4771 (0.58%), *Ralstonia* OTU1370 (1.08), and *Pseudomonas* OTU18020 (0.23%) were identified as core OTUs at salinity B. *Candidatus Bacilloplasma* OTU360 (1.80%), *Candidatus Bacilloplasma* OTU75 (1.20%), *Pseudomonas* OTU18484 (0.74%), *Candidatus Bacilloplasma* OTU18492 (0.46%) *Ralstonia* OTU1370 (1.34), and *Vibrio* OTU1121 (0.44%) were identified as core OTUs at salinity C. *Pseudomonas* OTU18484 (0.49%), *Ralstonia* OTU1370 (2.86%), and *Aeromonas* OTU3599 (0.23%) were identified as core OTUs at salinity D (Supplementary Table S5), which indicated that the core gut bacterial taxa of shrimp comprised more opportunistic pathogens under lower salinity culture conditions.

**Figure 4:**
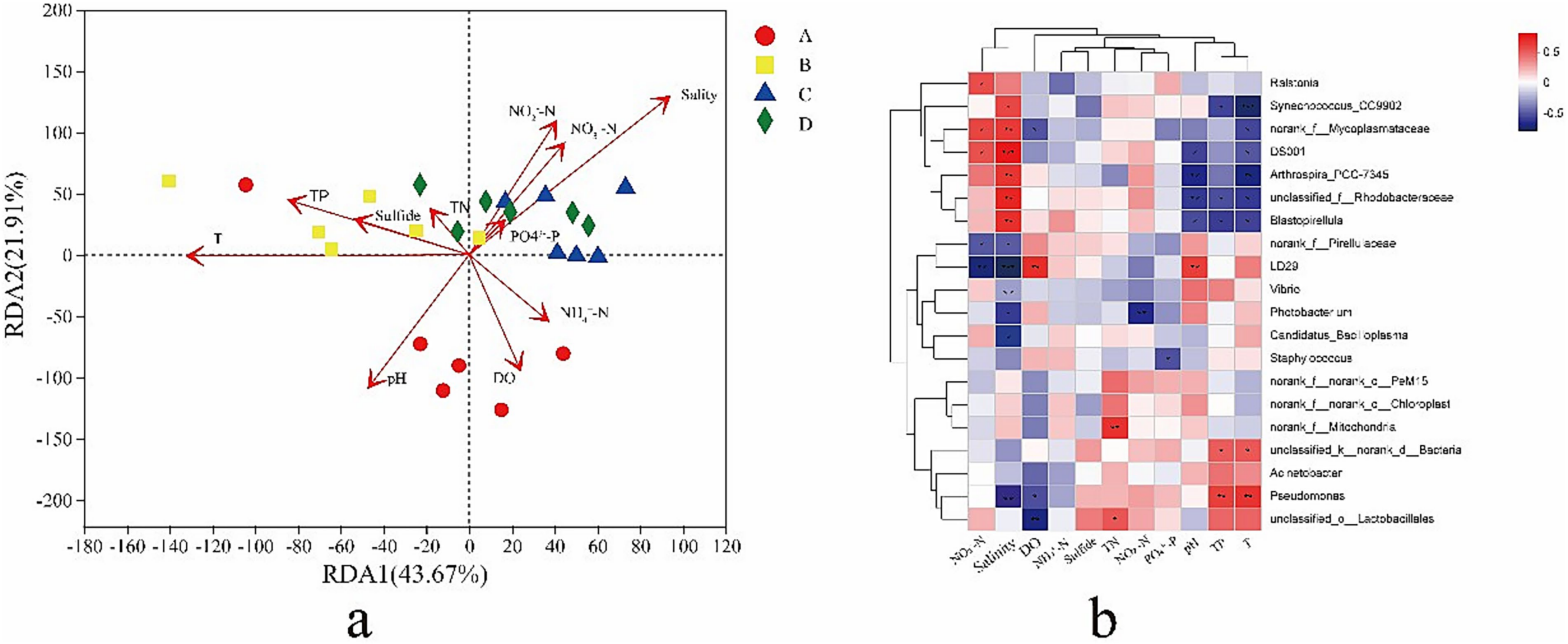
Redundancy analysis (RDA) based on the phylum data and environmental factors **(a)**. Arrows indicate the direction and magnitude of environmental factors related to microbial communities. The length of an arrow-line indicates the strength of relationship between microbial community and environmental variable. Dots of the same color represent the microbial communities in one group. A heat map showing the correlations between the top 20 bacterial genus in relative abundance and environmental factors **(b)**. A, B, C, D mean salinity 31 ± 0.85, 39 ± 1.23, 47 ± 0.62, and 55 ± 1.74.

An RDA diagram was constructed (Figure 4a), and axis 1 and axis 2 explained 43.67 and 21.91% of the variation in the data, respectively. After removing the redundant variables, 11 environmental characteristics were selected for RDA. Salinity (*R*^2^ = 0.6448, *p* = 0.002), T (temperature) (*R*^2^ = 0.3937, *p* = 0.012), pH (*R*^2^ = 0.33324, *p* = 0.029), NO_2_^−^-N (*R*^2^ = 0.4636, *p* = 0.041), and NO_3_^−^-N (R^2^ = 0.245, *p* = 0.051) were significantly correlated with the gut bacterial community structure of shrimp. The results indicated that salinity was the most important environmental factor affecting the structure of the gut bacterial community. A heat map revealed correlations between the 20 most abundant genera and environmental factors (Figure 4b). NO_3_^−^ - N was significantly positively correlated with *Ralstonia*, *norank_f_Mycoplasmataceae*, and *DS001* (*p* < 0.05) and significantly negatively correlated with *norank_f_Pirellulaceae* and *Sphingobium yanoikuyae* (*p* < 0.05). Salinity was significantly positively correlated with *Synechococcus_CC9902*, *norank_f_Mycoplasmataceae*, *DS001*, *Arthrospira_PCC-7345*, *unclassified_f_Rhodobacteraceae*, and *Blastopirellulaceae* (*p* < 0.05) and significantly negatively correlated with *norank_f_Pirellulaceae*, *Sphingobium yanoikuyae*, *Vibrio*, *Photobacterium*, *Candidatus_Bacilloplasma*, and *Pseudomonas* (*p* < 0.05). DO was significantly positively correlated with *Sphingobium yanoikuyae* (*p* < 0.05) and significantly negatively correlated with *norank_f_Mycoplasmataceae* (*p* < 0.05). TN was significantly positively correlated with *norank_f_Mitochondria* and *unclassified_o_Latobacillales* (*p* > 0.05). NO_2_^−^ - N was significantly negatively correlated with *Photobacterium* (*p* < 0.05). PO_4_^3−^ - P was significantly negatively correlated with *Staphylococcus* (*p* < 0.05). pH was only significantly positively correlated with *Sphingobium yanoikuyae* (*p* < 0.05) and significantly negatively correlated with *DS001*, *Arthrospira_PCC-7345*, *unclassified_f_Rhodobacteraceae*, and *Blastopirellula* (*p* < 0.05). TP was significantly positively correlated with *unclassified_k_norank_d_Bacteria* and *Pseudomonas* (*p* < 0.05) and significantly negatively correlated with *Synechococcus_CC9902*, *unclassified_f_Rhodobacteraceae*, and *Blastopirellula* (*p* < 0.05). T (temperature) was significantly positively correlated with *unclassified_k_norank_d_Bacteria* and *Pseudomonas* (*p* < 0.05) and significantly negatively correlated with *Synechococcus_CC9902*, *norank_f_Mycoplasmataceae*, *DS001*, *Arthrospira_PCC-7345*, and *Blastopirellula* (*p* < 0.05).

### 3.4 Functional differences in the gut bacterial community of shrimp

**Figure 5:**
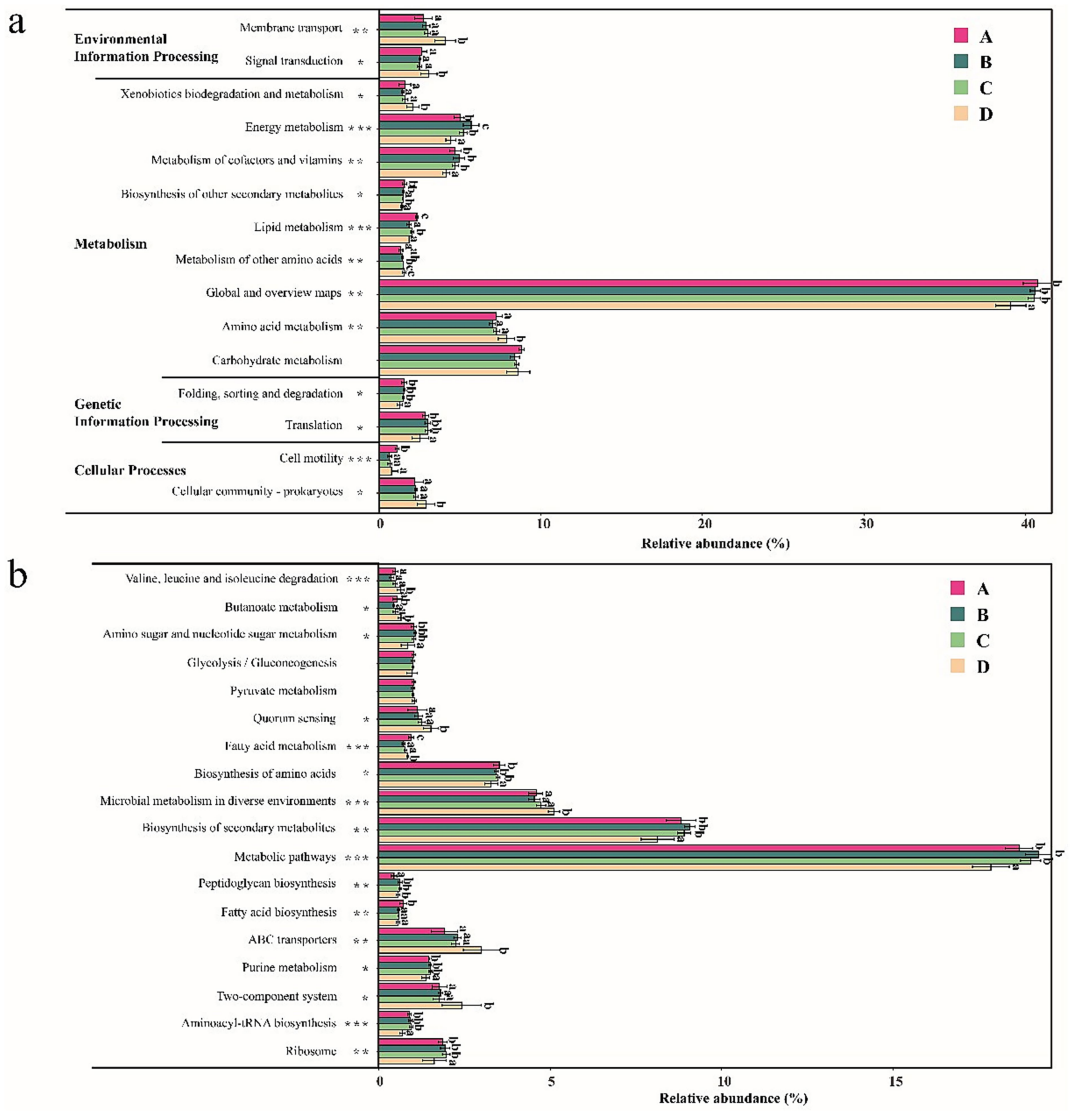
The difference in the abundance of KEGG functional pathways of gut bacterial community at level-2 **(a)** and level-3 **(b)**. A, B, C, D mean salinity 31 ± 0.85, 39 ± 1.23, 47 ± 0.62, and 55 ± 1.74. Different superscript letters indicate significant differences between the same column (*p* < 0.05).

**Figure 6:**
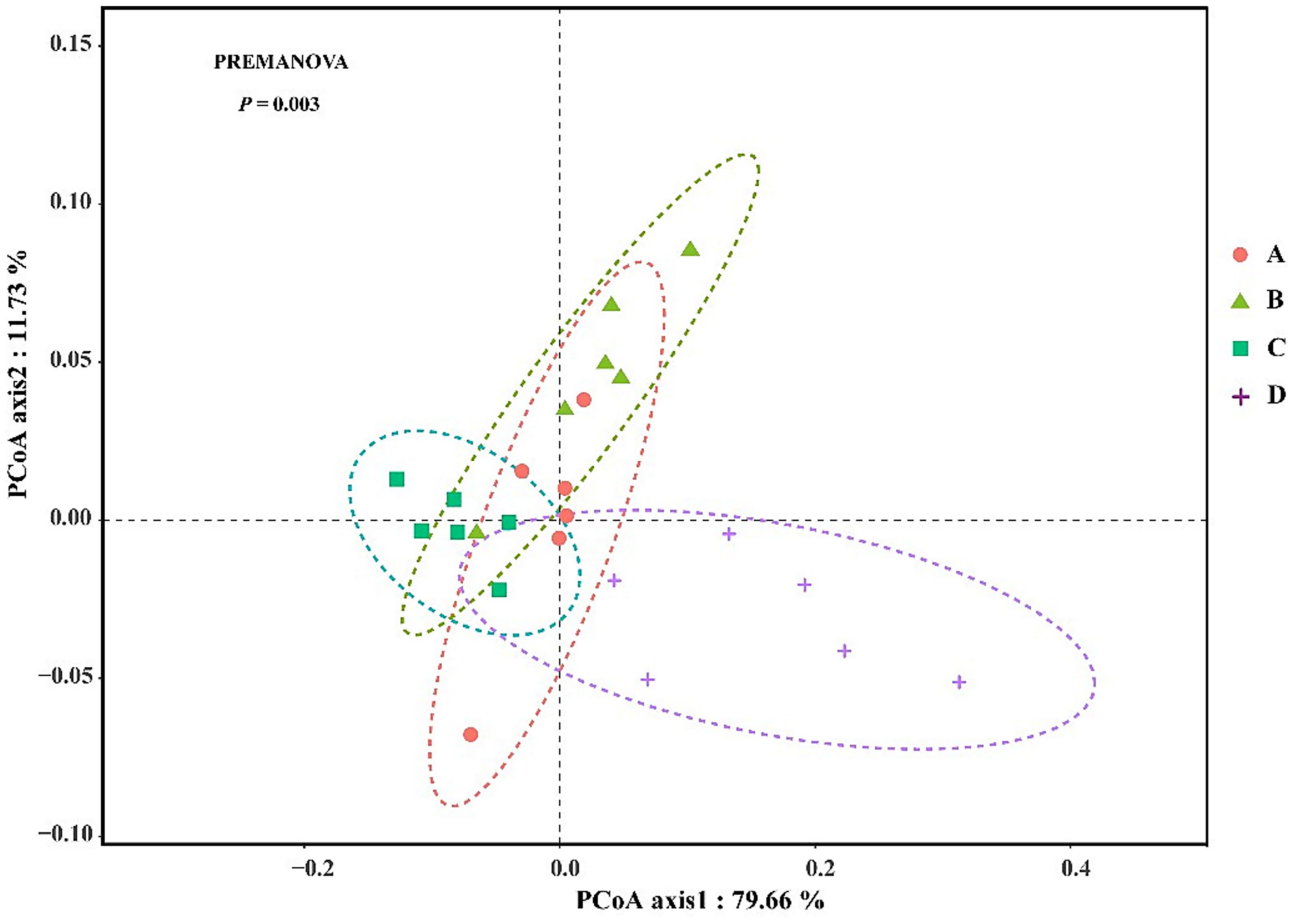
Principal coordinated analysis (PCoA) of the gut bacterial community function content similarity based on metagenomic functional predictions at level-3.

PICRUSt2 analysis was performed to predict the functions of the gut bacterial community of shrimp. Differences in the relative abundance of level 2 and level 3 functional pathways of the gut bacterial community based on the KEGG database are shown in Figure 5. As shown in Figure 5a, a total of 15 level 2 functional pathways were compared, and significant differences (*p* < 0.05) were observed in 14 functional pathways of the gut bacterial community of shrimp. The relative abundance of six functional pathways (Cellular community—prokaryotes, Amino acid metabolism, Metabolism of other amino acids, Xenobiotics biodegradation and metabolism, Signal transduction, and Membrane transport) was significantly higher at salinity D than at the other salinities (*p* < 0.05). No significant differences were observed among the three groups (*p* > 0.05). The relative abundance of seven functional pathways (Energy metabolism, Metabolism of cofactors and vitamins, Biosynthesis of other secondary metabolites, Lipid metabolism, Global and overview maps, Folding sorting and degradation, and Translation), was significantly lower at salinity D than at the other three salinities (*p* < 0.05). No significant differences were observed among the three other salinity groups (*p* > 0.05). A total of 18 level 3 functional pathways were compared, and significant differences (*p* < 0.05) were observed in 16 functional pathways of the gut bacterial community of shrimp (Figure 5b). The relative abundance of six functional pathways (Two-component system, ABC transporters, Microbial metabolism in diverse environments, Quorum sensing, Butanoate metabolism, and Valine leucine and isoleucine degradation) was significantly higher at salinity D than at the other three salinities (*p* < 0.05), and no significant differences were observed among the three salinity groups (*p* > 0.05). The abundance of seven functional pathways (Ribosome, Aminoacyl-tRNA biosynthesis, Purine metabolism, Metabolic pathways, Biosynthesis of secondary metabolites, Biosynthesis of amino acids, and Amino sugar and nucleotide sugar metabolism) was significantly lower at salinity D than at the other three salinities (*p* < 0.05); no significant differences were observed among the three groups (*p* > 0.05). The above results showed that the functions of the gut bacterial community significantly differed when the salinity was 55 ± 1.74. A PCoA analysis was conducted to analyze the functional content similarity of all samples (i.e., data explained 91.39% of the variation) (Figure 6). Significant separation was observed among samples at different salinities (*p* < 0.05). Salinity D samples were most clearly separated from the samples at the other three salinities. The results suggested that salinity is an important environmental factor affecting the functions of the gut bacterial community of shrimp.

### 3.5 Interspecific interactions of the gut microbiota in shrimp

**Figure 7:**
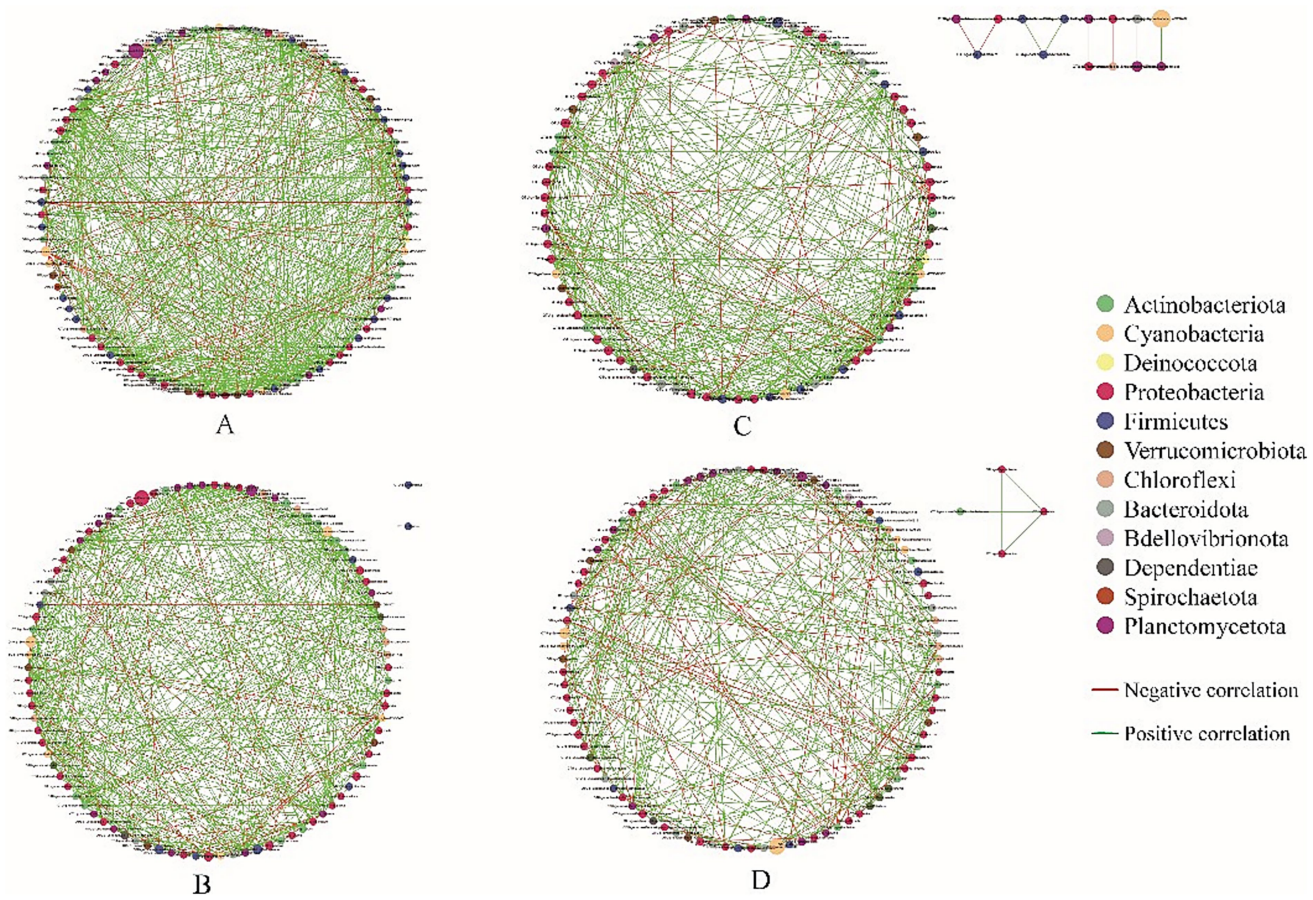
Co-occurrence networks analysis of the gut microbial community under different salinity levels. Each node represents one genus. Node colors indicate genus affiliated to different major phylum. The green edge indicates positive relationship between two individual nodes, whereas the red edge indicates negative relationship. The (**A–D**) represent the salinities 31 ± 0.85, 39 ± 1.23, 47 ± 0.62, and 55 ± 1.74, respectively.

Analysis of the structure of co-occurrence networks can provide insights into interactions among microbes (Barberán et al., 2012). To evaluate the effect of salinity on interspecific interactions of the gut bacterial community, the interspecies interaction network of the 100 most abundant genera was established. The network plots showed that the bacterial networks in the shrimp gut at salinities A and B were more complex and had higher interconnectedness than those at salinities C and D, as indicated by the greater number of connections and larger network size (Figure 7). This pattern was further confirmed by the topological properties, as the number of edges, the average number of neighbors, network density, and network diameter were higher in the shrimp gut at salinities A and B than at salinities C and D (Table 2). The percentage of positive associations decreased with salinity (Table 2).

**Table 2:** Topological parameters of co-occurrence network for gut microbial community at different salinity levels.

Ponds	A	B	C	D
Nodes	93	93	91	96
Edges	554	490	368	314
Cluster coefficient	0.28	0.26	0.24	0.21
Average number of neighbors	11.91	10.54	8.08	6.54
Network density	0.065	0.057	0.045	0.034
Network diameter	7	7	5	4
Positive association (%)	90.61	88.16	82.87	79.30
Negative association (%)	9.39	11.84	14.13	20.70

### 3.6 Assembly of the gut microbial community in shrimp

**Figure 8:**
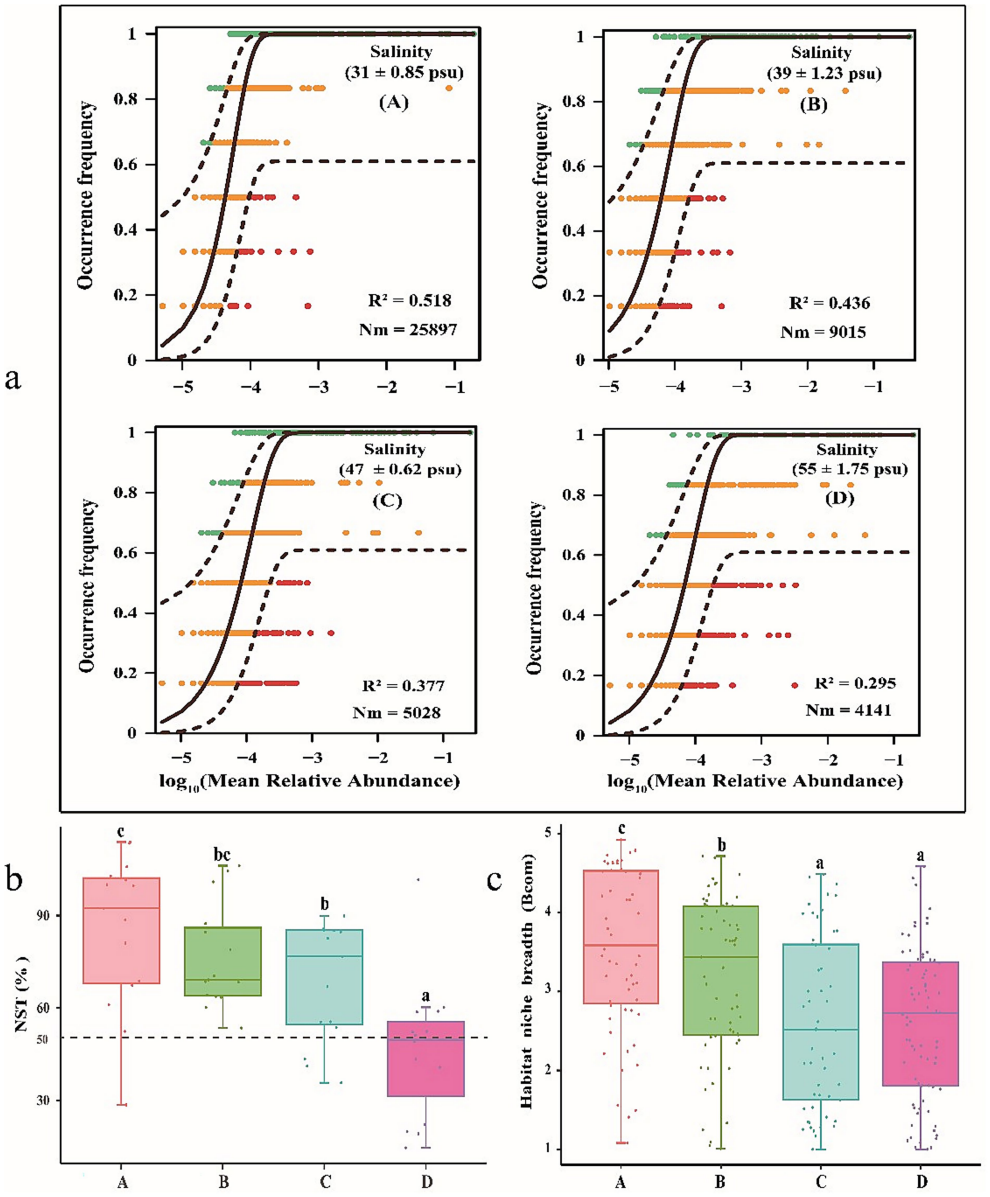
Relative contribution ratios of deterministic and stochastic processes on microbial community assembly in gut of shrimp. A, B, C, D mean salinity 31 ± 0.85, 39 ± 1.23, 47 ± 0.62, and 55 ± 1.74. The letters (**a-c**) indicate significant differences between the data groups.

The NCM quantified the relationship between the occurrence frequency of OTUs and their relative abundance (Figure 8a). The relative contribution of stochastic processes gradually decreased with salinity and explained 51.8, 43.6, 37.7, and 29.5% of the community variance at salinities A, B, C, and D, respectively. The NST was also used to quantify the relative contribution of stochastic and deterministic processes in gut bacterial community assembly (Figure 8b). The NST average value was above the 50% boundary point for the gut bacterial community at salinities A, B, and C, which was 85.18, 76.29, and 68.49%, respectively. This result indicated that the gut bacterial community at salinities A, B, and C was predominately regulated by stochastic processes. However, the NST average value of the gut bacterial community at salinity D was 46.26%, suggesting that deterministic processes had a marginally stronger effect than stochastic processes on the gut bacterial community at salinity D. These observations suggested that the strength of the effect of deterministic processes on the gut bacterial community of shrimp increased with salinity whereas that of stochastic processes decreased with salinity. In addition, community-level habitat niche breadths (Bcom) were also calculated to explore the relative importance of deterministic and stochastic processes in gut bacterial community assembly. The gut bacterial community had significantly wider niche breadths at salinity A than at other salinities (*p* < 0.05) (Figure 8c). The community-level habitat niche breadths (Bcom) decreased with salinity, which suggested that gut bacterial community assembly was more strongly affected by deterministic processes at higher salinity levels.

## 4 Discussion

The gut microbiota of shrimp plays a key role in maintaining host health and promoting disease resistance, and an increasing number of studies have examined the factors affecting the structure and function of the gut microbiota (Rooks and Garrett, 2016). The gut microbiota of shrimp is influenced not only by biological factors (Dai et al., 2017; Zheng et al., 2020; Zhou et al., 2020; Zhang et al., 2021) but also by abiotic factors such as salinity, temperature, and pH (Schmidt et al., 2015; Zhang M. et al., 2016; Hou et al., 2017; Huang et al., 2018). Therefore, understanding the composition and functions of the gut bacterial community in aquaculture systems and their relationships with environmental factors is critically important for improving the management of shrimp culture. The extensive farming of shrimp in the primary salt evaporation ponds of the salterns in the coastal areas of the Yellow and Bohai Seas is critically important for increasing the natural resource utilization and economic benefits of these regions. However, few studies have reported the characteristics of the gut bacterial community of shrimp cultured in these hypersaline environments.

Microbial diversity, especially gut microbial diversity, is a key characteristic affecting host health (Ren et al., 2019). In this study, alpha diversity (Shannon index, Simpson index, Chao1 index, and ACE index) decreased with salinity. This result is consistent with the findings of previous studies (Cornejo-Granados et al., 2018; Hou et al., 2020). The results of PCoA and a hierarchical clustering tree showed that the threshold effects of salinity on bacterial community structure and phylogenetic relationships were observed when the salinity exceeded 39; these findings indicate that the salinity is a critical factor influencing the bacterial community structure. Previous studies have shown that Proteobacteria, Firmicutes, Bacteroides, and Actinobacteria are the predominant phyla in the gut bacterial community of *P. vannamei* in hyposaline environments (Duan et al., 2018; Li et al., 2018; Gao et al., 2019). Proteobacteria, as a highly adaptable phylum, plays a key role in nutrient acquisition and osmotic regulation. For instance, certain members of *Gammaproteobacteria* can synthesize compatible solutes (e.g., betaine and ectoine) to help the host maintain cellular osmotic balance under high salinity stress (Imhoff et al., 2021). Bacteroidetes contribute to nutrient acquisition via polysaccharide degradation enzymes. For instance, *Bacteroides thetaiotaomicron* is considered as the best degrader of polysaccharides (Li et al., 2021). Firmicutes, particularly lactic acid bacteria (LAB) belonging to this phylum, contribute significantly to intestinal health. LAB can produce lactic acid and bacteriocins, which inhibit the colonization of pathogenic bacteria (e.g., *Vibrio* spp.) by lowering the intestinal pH and competing for ecological niches (Yang et al., 2008). Actinobacteria, as a phylum with diverse metabolic capabilities, contribute to both nutrient metabolism and immune enhancement. Many Actinobacteria species can synthesize antibiotics and enzymes (e.g., proteases and lipases) that inhibit pathogenic microorganisms and aid in the digestion of proteins and lipids, which are essential for shrimp growth under stressful hypersaline conditions. For instance, *Streptomyces* are soil dwelling bacteria and characterized by their remarkable capacity for sporulation and biosynthesis of diverse secondary metabolites, particularly antibiotics (Jones, 2023). Salinity plays a dominant role in shaping the intestinal microbiota of shrimp which change the osmotic pressure and ion concentrations in the gut, determining the survival of microorganisms and enhancing digestion and immunity (Gou et al., 2018; Chen et al., 2022). In our study, the predominant phyla in the gut bacterial community of *P. vannamei* under hypersaline environments were Cyanobacteria, Proteobacteria, Planctomycetes, Firmicutes, and Actinobacteria. This suggests that salinity plays an important role in the gut bacterial composition of *P. vannamei*. The relative abundances of some opportunistic pathogens were significantly lower at salinities of 47 (C) and 55 (D), such as *Vibrio*, *Pseudomonas*, *Candidatus_Bacilloplasma*, and *Photobacterium*, than at 31 (A) and 39 (B). The excessive growth of opportunistic pathogens in the gut of aquatic animals can contribute to disease (Pérez et al., 2010; Xing et al., 2013; Huang et al., 2020). For example, the overgrowth of *Vibrio*, *Pseudomonas*, *Candidatus_Bacilloplasma*, and *Photobacterium* can have pathogenic effects on aquatic animals (Soto-Rodriguez et al., 2015; Liu et al., 2016; Hou et al., 2018a; Xin et al., 2018). Bacterial diseases, mainly caused by the *Vibrio* genus, are considered as the most serious threat to shrimp aquaculture. For instance, the common pathogenic *Vibrio* species: *Vibrio alginolyticus*, *Vibrio harveyi*, *Vibrio parahaemolyticus*, usually induced the pale hepatopancreas, empty gut, and sloughing of the epithelial cells of the hepatopancreas tubule and massive hemolytic infiltration (Baker-Austin et al., 2018; Lee et al., 2015; Zhang et al., 2023). *Pseudomonas aeruginosa* threatens whiteleg shrimp by causing black gill disease and shell ulcers via toxin production (LasB, pyocyanin), suppressing immunity (Ramalingam and Ramarani, 2007). *Photobacterium* may cause symptoms such as pale hepatopancreas and whitish body coloration in shrimp, ultimately leading to mortality (Liu et al., 2016). Study has revealed significant intestinal proliferation of *Candidatus Bacilloplasma* in *P. vannamei* affected by White Feces Syndrome (WFS), suggesting its potential role as a key etiological agent in WFS pathogenesis (Hou et al., 2018b). This means that the hypersaline environment could reduce the mortality rate of shrimp caused by opportunistic bacterial pathogens to some degree.

The core microbiota and their compositions are the characteristic microbiome of a microbial community and play a key role in bacterial community functions (Shade and Handelsman, 2012). Core microbiota in shrimp intestines may aid nutrient digestion by secreting enzymes to break down complex carbohydrates and proteins (Tao et al., 2020), enhance immunity via competing with pathogens and stimulating antimicrobial peptide production and maintain gut homeostasis by regulating pH and redox balance, supporting host health and environmental adaptation (Macke et al., 2017). Identification of the core microbiota associated with a host is essential for clarifying the key microbial metabolic functions of the host (Mueller and Sachs, 2015). In general, polysaccharide digestion, essential amino acid biosynthesis, SCFA production and lipid metabolism are all essential for hosts (Song et al., 2021). Some studys foud that microorganisms such as some species of *Vibrio* and *Flavobacterium* are often present in the early larval stage of shrimp. For example, studies by Holt et al. (2020) have shown that in the gastrointestinal tract of giant tiger prawns and Pacific white shrimp, γ-Proteobacteria, including *Vibrio*, are dominant in the early stages. As the shrimp grow into the juvenile stage, the proportion of some beneficial bacteria like *Lactobacillus* may gradually increase, which can help with the digestion and immune function of the shrimp (Xia et al., 2020). During the adult stage, the core microbiome may be more complex, with the co-existence of a variety of bacteria involved in nutrient metabolism, such as certain members of the Bacteroidetes and Actinobacteria (Li et al., 2021, Jones, 2023). Studies have shown that variation in core gut microbes largely depends on the host environment (Mortzfeld et al., 2016). In our study, the composition of the core gut microbes of shrimp cultured at different salinities was compared. The core microbes significantly differed at the four salinity levels, and only 13 core microorganisms were shared among groups at various salinities. Part of the core gut microbes of the host can be identified early in life because of their significant contribution to basic gut microbial functions (Shade and Handelsman, 2012). The other part may be acquired through the deterministic process of colonization of the gut with specific microbes from the environment, which perform specific functions in the host (Weigel, 2020). The above results indicated that salinity strongly affected the composition of the core microorganisms in the gut of shrimp. In general, the core gut microbes are considered beneficial to host health (Walter and Ley, 2011). However, the results of this study showed that the core gut microbes of shrimp cultured at salinities of 31 and 39 3 contained more opportunistic pathogens than those at salinities of 47 and 55.

Salinity and temperature are key environmental factors that shape gut microbiota composition and function through direct and host-mediated mechanisms (Staley et al., 2015; Sunagawa et al., 2015; Li et al., 2018). Salinity influences gut microbiota by altering osmotic pressure in the intestinal lumen. High salinity selects for osmotolerant taxa (e.g., *Vibrio*, *Pseudorhodobacter*) while suppressing osmosensitive species like *Cetobacterium* in *Gymnocypris przewalskii* (Wang et al., 2022). This shift reduces diversity and disrupts metabolic pathways, such as osmoregulation via compatible solute accumulation. Temperature directly impacts microbial enzyme activity and membrane fluidity. In ectotherms like European sea bass (*Dicentrarchus labrax*), seasonal temperature drops correlate with increased pathogenic *Vibrio* abundance and altered skin/intestinal microbiota structure (Mokrani et al., 2019). Temperature also modulates host metabolism, affecting nutrient availability and indirectly driving shifts in fermentation pathways (e.g., short-chain fatty acid production). Extreme temperatures induce heat-shock proteins and immune activation, compromising gut barrier integrity and promoting dysbiosis. These factors often act synergistically. For instance, in aquaculture, combined salinity and temperature fluctuations exacerbate microbial imbalance, increasing disease susceptibility. Collectively, salinity and temperature serve as ecological filters, reshaping gut microbiota to influence host health and adaptive fitness. Some researchers indicate that salinity was more important environmental factor affecting the global microbial community distribution patterns than temperature (Lozupone and Knight, 2007; Auguet et al., 2010; Wang et al., 2011). Similarly, salinity was more important than temperature on the gut bacterial community of shrimp in our study. There was a negative correlation between salinity and the abundance of some opportunistic pathogens, such as *Vibrio*, *Photobacterium*, *Candidatus_Bacilloplasma*, and *Pseudomonas* (Figure 4b), which was consistent with the results of a previous study (Bauer et al., 2021). In addition to salinity and temperature, pH, NO_2_^−^ - N, and NO_3_^−^ - N concentrations were significantly correlated with gut bacterial community structure, and similar observations were made in other studies (Hou et al., 2017; Huang et al., 2018). However, the gut bacterial community of shrimp is affected not only by the surrounding environment but also by the hosts (Pérez et al., 2010; Sullam et al., 2012). Additional in-depth studies are needed to clarify the interactions between environmental pressures and host selective mechanisms. Salinity and temperature have a significant effect on microbial community functions (Székely et al., 2013; Sunagawa et al., 2015). Some previous studies have shown that temperature plays a greater role than salinity in affecting the functional composition of microorganisms in the marine environment (Sunagawa et al., 2015). In our study, salinity was a key factor affecting the functions of the gut bacterial community in shrimp. The threshold effects of salinity on the functions of the gut bacterial community of shrimp were observed at the salinity of 55 had a major effect on the functions of the gut bacterial community of shrimp. Similar results were obtained by PCoA. This finding indicated that salinity affected the functions of the bacterial community of shrimp in hypersaline environments. The gut microbiota comprises a variety of species that interact with each other and form a complex ecological network characterized by different types of interactions, such as cooperative, competitive, and predatory interactions (Deng et al., 2012). Co-occurrence networks can provide valuable insights into biological interactions within the microbial community (Williams et al., 2014). Generally, complex networks with high connectivity are more robust against external disturbances and more active than simple networks (Lu et al., 2013; Santolini and Barabási, 2018). In this study, the gut bacterial networks of shrimp were more complex and better connected in lower salinity environments than those in higher salinity environments, suggesting that the gut microbial ecosystem is more stable and active in hyposaline environments than in hypersaline environments. In addition, the percentage of positive associations decreased with salinity, which was consistent with the results of a previous study (Hou et al., 2020), suggesting that there was a higher degree of cooperative activities in the shrimp gut at lower salinity (Montoya et al., 2006).

In general, the microbial community assembly is driven by deterministic and stochastic process (Hanson et al., 2012). Deterministic processes refer to ecological assembly mechanisms driven by environmental filtering (e.g., salinity, pH, nutrients) and biological interactions (e.g., competition, predation), leading to predictable community structures and stochastic processes involve random events (e.g., dispersal limitation, ecological drift) that contribute to unpredictable variations in microbial communities (Dini-Andreote et al., 2015). Deterministic processes regulate the fitness of microbial communities, thereby determining species composition and relative abundance, whereas stochastic processes induce unpredictable alterations in community structure. Ultimately, these two distinct processes collectively govern the functional roles of microbial communities in biogeochemical cycles (Tilman, 2004; Zhang W. et al., 2016). In the present study, salinity had an important effect on the assembly of the gut bacterial community of shrimp, mainly by affecting the balance between deterministic and stochastic processes. The R^2^ values of the NCMs decreased with salinity, suggesting that the relative contribution of stochastic processes in the gut bacterial community of shrimp decreased with salinity. The NST index also revealed that the relative contribution of deterministic processes compared with stochastic processes in the gut bacterial community of shrimp increased with salinity, likely because the greater environmental heterogeneity at high salinity exposes the gut bacterial community to a wider range of filters, which increases the strength of deterministic processes of environmental selection (Vellend et al., 2014). Species with a wider niche range are considered generalists that exhibit greater metabolic plasticity and are less affected by environmental factors due to their higher environmental tolerances (Pandit et al., 2009; Li et al., 2019). Therefore, we characterized the habitat niche breadths of the gut bacterial community of shrimp and found that niche breadths decreased with salinity, suggesting that the assembly of the gut bacterial community in shrimp was more strongly affected by deterministic processes as salinity increased. A niche refers to the functional role and habitat conditions that a microorganism occupies within an ecosystem, including its resource use and environmental tolerances. In the gut microbiota, niche differentiation (e.g., oxygen gradients, nutrient availability) shapes microbial diversity and stability. In this study, the gut bacterial community had significantly wider niche breadths at salinity A than at other higher salinities, which suggested salinity fluctuations may alter niche availability, favoring halotolerant taxa and restructuring bacterial community structure. The results further demonstrated that the gut bacterial community in shrimp was increasingly governed by deterministic processes with elevated salinity levels.

This study highlights gut microbiota modulation as a viable strategy to improve shrimp health in hypersaline environment, with specific relevance to large ponds in salt pan. Future research should focus on: (1) developing locally-adapted probiotics from indigenous halotolerant strains, (2) deciphering host-microbiota metabolic crosstalk via multi-omics under hypersalinity, and (3) creating salinity-responsive management protocols. Practical implementation requires farmer-friendly delivery systems coupled with ecological monitoring. Such microbiome interventions may offer sustainable, chemical-free solutions for the shrimp farming in large ponds of salt pan.

## 5 Conclusion

Changes in the characteristics of the gut microbiota in *P. vannamei* with salinity were investigated in the primary salt evaporation ponds for the salterns in northern China. The results demonstrated that salinity significantly affected the gut bacterial community of shrimp. Alpha diversity indexes of the gut bacterial community in shrimp decreased with salinity. Potential opportunistic bacterial pathogens decreased significantly in hypersaline environments. Furthermore, the gut bacterial community structure differed significantly between salinities 31–39 and 47–55, the predicted functions were distinct at salinities of 31–47 and 55. Stochastic and deterministic processes jointly contributed to the assembly of the gut bacterial community of shrimp; however, the relative importance of stochastic processes decreased with salinity. Our findings provide new insights into the characteristics of the gut bacterial community of shrimp in hypersaline environments and contribute to the improvement of farming health management in hypersaline ponds.

## Data Availability

The datasets presented in this study can be found in online repositories. The names of the repository/repositories and accession number(s) can be found in the article/Supplementary material.
