# Supplementary material for: Characteristics of gut microbiota in Penaeus vannamei shifted with elevated salinity
Source: Front Microbiol. 2025 Sep 17;16:1665547. doi: 10.3389/fmicb.2025.1665547 (PMC12486418; doi:10.3389/fmicb.2025.1665547)
Supplement: Supplementary file 1 [file Data_Sheet_1.docx]

Supplemental Table S1 Production of *Penaeus vannamei* in the ponds with different salinity

| Salinity | Shrimp production (kg / ha) |
| --- | --- |
| A (31 ± 0.85) | 847.98 ± 19.61 |
| B (39 ± 1.23) | 752.11 ± 14.36 |
| C (47 ± 0.62) | 603.33 ± 28.37 |
| D (55 ± 1.74) | 263.04 ± 31.25 |

Supplemental Table S2 Physical and chemical properties of water

|  | NH_4_^+^-N | NO_2_^-^-N | NO_3_^-^-N | TN | Sulfide | TP | PO_4_^3-^-P | Salinity | pH | DO | T |
| --- | --- | --- | --- | --- | --- | --- | --- | --- | --- | --- | --- |
| Water | (mg L^-1^) | (mg L^-1^) | (mg L^-1^) | (mg L^-1^) | (mg L^-1^) | (mg L^-1^) | (mg L^-1^) | (g L^-1^) |  | (mg L^-1^) | (℃) |
| ASw_1 | 0.1623 | 0.040 | 0.2438 | 3.2978 | 0.1190 | 0.5076 | 0.0173 | 30.97 | 8.07 | 9.45 | 25.2 |
| ASw_2 | 0.2280 | 0.049 | 0.2237 | 5.3796 | 0.1273 | 0.4196 | 0.0344 | 32.06 | 8.1 | 10.93 | 25.5 |
| ASw_3 | 0.1650 | 0.032 | 0.2331 | 2.3055 | 0.1203 | 0.5211 | 0.0153 | 31.22 | 8.04 | 11.65 | 25.9 |
| ASw_4 | 0.1610 | 0.025 | 0.2852 | 2.3880 | 0.1177 | 0.4670 | 0.0392 | 31.75 | 8.12 | 12.18 | 26.1 |
| ASw_5 | 0.2253 | 0.019 | 0.2390 | 4.6729 | 0.1853 | 0.4196 | 0.0222 | 31.57 | 8 | 9.64 | 25.7 |
| ASw_6 | 0.2423 | 0.0184 | 0.2451 | 3.8729 | 0.2041 | 0.4526 | 0.0235 | 31.24 | 8.23 | 9.04 | 26.3 |
| BSw_1 | 0.1830 | 0.064 | 0.3953 | 3.7837 | 0.1730 | 0.4399 | 0.0250 | 38.65 | 7.94 | 9.63 | 25.8 |
| BSw_2 | 0.1924 | 0.053 | 0.4935 | 3.7837 | 0.1387 | 0.5618 | 0.0275 | 39.77 | 7.93 | 9.62 | 25.5 |
| BSw_3 | 0.1726 | 0.080 | 0.2544 | 3.8308 | 0.2533 | 0.4128 | 0.1295 | 39.71 | 7.96 | 10.31 | 25.5 |
| BSw_4 | 0.1875 | 0.060 | 0.2248 | 4.4167 | 0.1747 | 0.3993 | 0.0273 | 39.75 | 7.91 | 9.98 | 25.3 |
| BSw_5 | 0.1844 | 0.059 | 0.5302 | 4.4668 | 0.1860 | 0.4534 | 0.0256 | 39.79 | 7.93 | 9.58 | 25.4 |
| BSw_6 | 0.1941 | 0.069 | 0.5289 | 4.5581 | 0.1901 | 0.4236 | 0.0268 | 40.05 | 7.68 | 9.46 | 25.3 |
| CSw_1 | 0.1850 | 0.050 | 0.3953 | 4.8790 | 0.1440 | 0.4027 | 0.0331 | 46.63 | 8.01 | 8.06 | 24.4 |
| CSw_2 | 0.2137 | 0.066 | 0.2651 | 4.6729 | 0.1350 | 0.4027 | 0.0187 | 47.34 | 7.95 | 8.15 | 24.5 |
| CSw_3 | 0.1543 | 0.048 | 0.3066 | 6.4750 | 0.1023 | 0.4027 | 0.0374 | 46.61 | 7.95 | 7.87 | 24.5 |
| CSw_4 | 0.1715 | 0.054 | 0.4533 | 3.8249 | 0.1023 | 0.4128 | 0.0268 | 46.69 | 7.96 | 7.2 | 24.5 |
| CSw_5 | 0.1537 | 0.064 | 0.7126 | 3.0711 | 0.1333 | 0.7886 | 0.0345 | 46.71 | 7.95 | 7.25 | 24 |
| CSw_6 | 0.1641 | 0.059 | 0.7109 | 3.0689 | 0.1356 | 0.7468 | 0.0312 | 47.03 | 7.88 | 7.31 | 24.3 |
| DSw_1 | 0.1936 | 0.058 | 0.6569 | 6.3336 | 0.2117 | 0.6058 | 0.0240 | 54.97 | 7.96 | 6.75 | 26.5 |
| DSw_2 | 0.1788 | 0.051 | 0.4379 | 5.5150 | 0.2280 | 0.6159 | 0.0298 | 53.99 | 7.94 | 7.78 | 27.1 |
| DSw_3 | 0.1785 | 0.064 | 0.2461 | 5.3737 | 0.2127 | 0.5922 | 0.0337 | 49.69 | 7.96 | 7.28 | 26.8 |
| DSw_4 | 0.1784 | 0.081 | 0.2130 | 6.2276 | 0.1537 | 0.6769 | 0.2399 | 54.99 | 7.97 | 7.45 | 27 |
| DSw_5 | 0.1962 | 0.069 | 0.1929 | 4.5257 | 0.1947 | 0.6599 | 0.0309 | 55.00 | 7.97 | 7.98 | 27.2 |
| DSw_6 | 0.1985 | 0.073 | 0.1908 | 4.4356 | 0.1899 | 0.6853 | 0.0389 | 55.49 | 8.01 | 7.86 | 26.3 |
| *P* value | 0.014 | 0.01 | 0.01 | 0.003 | 0.001 | 0.012 | 0.014 | 0.000 | 0.001 | 0.001 | 0.000 |

Supplemental Table S3 Sequence numbers and bacterial OTUs for every sample of gut bacterial community in shrimp

| Sample | Read numbers | Number of OTUs |
| --- | --- | --- |
| ASc_1 | 35873 | 720 |
| ASc_2 | 36344 | 350 |
| ASc_3 | 55548 | 1046 |
| ASc_4 | 53729 | 988 |
| ASc_5 | 60932 | 797 |
| ASc_6 | 45236 | 852 |
| BSc_1 | 55604 | 712 |
| BSc_2 | 59137 | 1041 |
| BSc_3 | 51605 | 991 |
| BSc_4 | 55605 | 1309 |
| BSc_5 | 62401 | 1407 |
| BSc_6 | 52304 | 1125 |
| CSc_1 | 50242 | 659 |
| CSc_2 | 50314 | 739 |
| CSc_3 | 48530 | 1102 |
| CSc_4 | 50704 | 623 |
| CSc_5 | 48490 | 1372 |
| CSc_6 | 48953 | 1056 |
| DSc_1 | 53780 | 1406 |
| DSc_2 | 61129 | 779 |
| DSc_3 | 109516 | 601 |
| DSc_4 | 64704 | 1055 |
| DSc_5 | 148098 | 1476 |
| DSc_6 | 112340 | 561 |


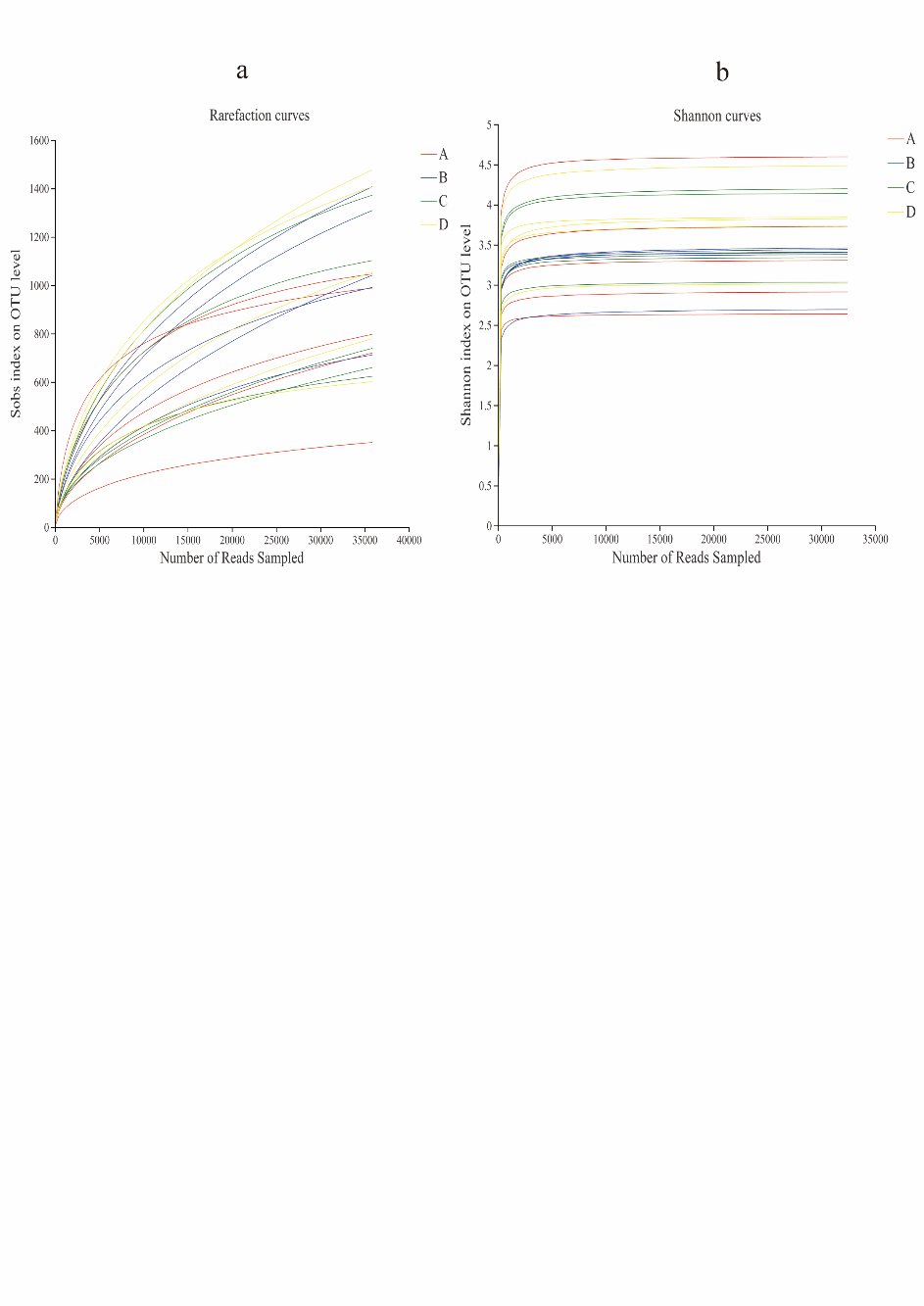


Supplemental Figure S1. Analysis of rarefaction curves. Rarefaction curve (a); Shannon-Wiener curves (b).


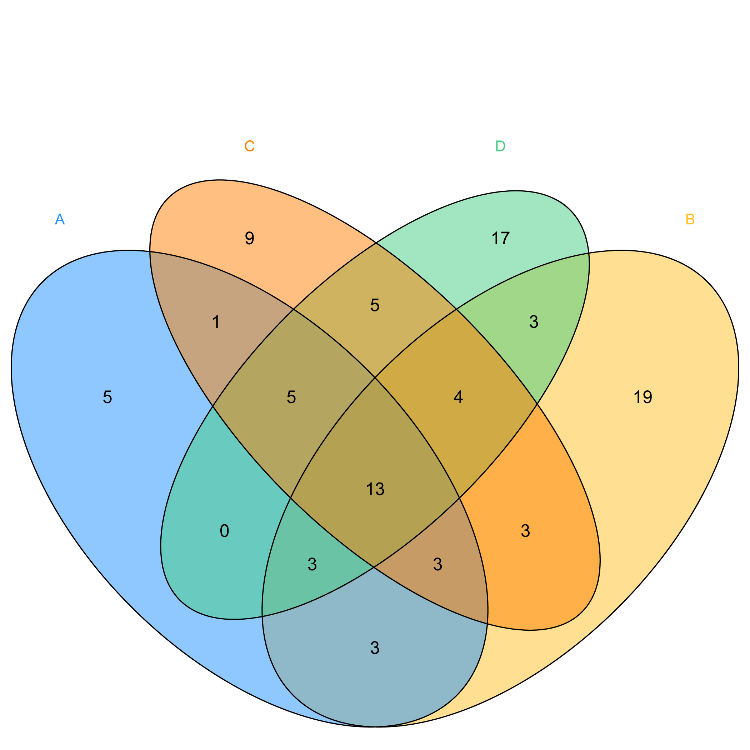


Supplemental Figure S2. The Venn diagram showing the unique and shared core OTUs of the gut bacterial community in shrimp at four salinities.

Supplemental Table S4 Taxonomic composition 10 shared core OTUs of the gut bacterial community in shrimp at four salinities

|  |  | Relative abundance (%) | | | |
| --- | --- | --- | --- | --- | --- |
| Shared core OTU | Genus | A | B | C | D |
| OTU1385 | *Rhodococcus* | 0.99 | 1.08 | 1.29 | 0.85 |
| OTU47 | *Pseudomonas* | 0.89 | 1.23 | 2.64 | 1.05 |
| OTU16545 | *Mycobacterium* | 1.25 | 2.56 | 0.93 | 1.85 |
| OTU11937 | *uncultured Planctomycetaceae bacterium* | 1.80 | 0.24 | 0.56 | 0.63 |
| OTU5407 | *uncultured Actinomycetales bacterium* | 1.86 | 1.75 | 0.93 | 1.76 |
| OTU24481 | *Delftia tsuruhatensis* | 0.99 | 0.79 | 0.27 | 1.01 |
| OTU5399 | *Planctomycetaceae bacterium D2* | 18.15 | 5.82 | 4.32 | 3.42 |
| OTU11967 | *Blastopirellula* | 0.65 | 0.47 | 2.26 | 1.37 |
| OTU1044 | *Synechococcus_CC9902* | 12.36 | 7.23 | 12.18 | 29.11 |
| OTU8993 | *uncultured Pirellulaceae bacterium* | 1.19 | 0.21 | 0.48 | 0.63 |
| OTU1370 | *Ralstonia* | 2.69 | 1.08 | 1.34 | 2.86 |
| OTU1207 | *Sphingobium yanoikuyae* | 2.98 | 1.36 | 2.21 | 1.62 |
| OTU5318 | *Microbacteriaceae bacterium CL-Dokdo102* | 0.38 | 1.34 | 1.39 | 3.16 |

Supplemental Table S5 Composition of gut core bacterial taxa and opportunistic pathogens in shrimp.

| A (31 ± 0.85) | | | | B (39 ± 1.23) | | | | | |
| --- | --- | --- | --- | --- | --- | --- | --- | --- | --- |
| Phylum | Genus | OTU ID | mean (%) | Phylum | | Genus | OTU ID | | mean (%) |
| Planctomycetota | norank_f__Pirellulaceae | OTU5399 | 18.15 | Proteobacteria | | Pseudomonas | OTU18484 | | 16.94 |
| Planctomycetota | norank_f__Pirellulaceae | OTU1056 | 14.11 | Cyanobacteria | | Synechococcus_CC9902 | OTU1044 | | 7.23 |
| Cyanobacteria | Synechococcus_CC9902 | OTU1044 | 12.36 | Planctomycetota | | norank_f__Pirellulaceae | OTU5399 | | 5.82 |
| Proteobacteria | Vibrio | OTU1121 | 4.98 | Cyanobacteria | | norank_f__norank_o__Chloroplast | OTU1151 | | 4.59 |
| Cyanobacteria | norank_f__norank_o__Chloroplast | OTU1151 | 4.26 | Firmicutes | | Candidatus_Bacilloplasma | OTU75 | | 4.43 |
| Verrucomicrobiota | LD29 | OTU1207 | 2.98 | Firmicutes | | unclassified_o__Lactobacillales | OTU24311 | | 4.39 |
| Proteobacteria | Ralstonia | OTU1370 | 2.69 | Proteobacteria | | Halomonas | OTU25125 | | 3.02 |
| Proteobacteria | Photobacterium | OTU1254 | 2.34 | Proteobacteria | | Comamonas | OTU24520 | | 2.61 |
| Actinobacteriota | norank_f__norank_o__PeM15 | OTU5407 | 1.86 | Planctomycetota | | norank_f__Pirellulaceae | OTU1056 | | 2.35 |
| Planctomycetota | norank_f__Pirellulaceae | OTU11937 | 1.80 | Proteobacteria | | Acinetobacter | OTU24502 | | 1.98 |
| Proteobacteria | norank_f__Mitochondria | OTU1369 | 1.23 | Actinobacteriota | | norank_f__norank_o__PeM15 | OTU5407 | | 1.75 |
| Planctomycetota | norank_f__Pirellulaceae | OTU8993 | 1.19 | Proteobacteria | | norank_f__Mitochondria | OTU1369 | | 1.62 |
| Firmicutes | Staphylococcus | OTU18163 | 1.17 | Cyanobacteria | | norank_f__norank_o__Chloroplast | OTU25168 | | 1.58 |
| Actinobacteriota | Rhodococcus | OTU1385 | 0.99 | Proteobacteria | | Acinetobacter | OTU17973 | | 1.45 |
| Proteobacteria | Delftia | OTU24481 | 0.99 | Actinobacteriota | | DS001 | OTU5318 | | 1.34 |
| Cyanobacteria | norank_f__norank_o__Chloroplast | OTU25168 | 0.76 | Actinobacteriota | | hodococcus | OTU1385 | | 1.30 |
| Firmicutes | Candidatus_Bacilloplasma | OTU18492 | 0.75 | Proteobacteria | | Acinetobacter | OTU12281 | | 1.23 |
| Proteobacteria | unclassified_c__Gammaproteobacteria | OTU8977 | 0.66 | Proteobacteria | | Photobacterium | OTU1254 | | 1.11 |
| Planctomycetota | Blastopirellula | OTU11967 | 0.65 | Proteobacteria | | Ralstonia | OTU1370 | | 1.08 |
| Proteobacteria | Acinetobacter | OTU17973 | 0.63 | Firmicutes | | Halolactibacillus | OTU24172 | | 1.02 |
| Proteobacteria | Vibrio | OTU267 | 0.58 | Proteobacteria | | Stenotrophomonas | OTU1259 | | 0.88 |
| Proteobacteria | Legionella | OTU4426 | 0.54 | Proteobacteria | | norank_f__Mitochondria | OTU11990 | | 0.80 |
| Bdellovibrionota | norank_f__norank_o__0319-6G20 | OTU4431 | 0.53 | Proteobacteria | | Delftia | OTU24481 | | 0.79 |
| Firmicutes | Staphylococcus | OTU8952 | 0.47 | Firmicutes | | Candidatus_Bacilloplasma | OTU18492 | | 0.74 |
| Cyanobacteria | Arthrospira_PCC-7345 | OTU10927 | 0.44 | Actinobacteriota | | ML602J-51 | OTU5336 | | 0.72 |
| Cyanobacteria | Synechocystis_PCC-6803 | OTU5340 | 0.44 | Proteobacteria | | Vibrio | OTU1121 | | 0.65 |
| Verrucomicrobiota | unclassified_o__Chlamydiales | OTU18200 | 0.41 | Verrucomicrobiota | | LD29 | OTU1207 | | 0.48 |
| Actinobacteriota | Mycobacterium | OTU16545 | 0.40 | Planctomycetota | | Blastopirellula | OTU11967 | | 0.47 |
| Actinobacteriota | DS001 | OTU5318 | 0.38 | Verrucomicrobiota | | unclassified_o__Chlamydiales | OTU18200 | | 0.45 |
| Verrucomicrobiota | Terrimicrobium | OTU5332 | 0.28 | Actinobacteriota | | norank_f__norank_o__PeM15 | OTU18328 | | 0.43 |
| Proteobacteria | Pseudomonas | OTU47 | 0.23 | unclassified_k__norank_d__Bacteria | | unclassified_k__norank_d__Bacteria | OTU15715 | | 0.38 |
| Proteobacteria | Stenotrophomonas | OTU1259 | 0.21 | Actinobacteriota | | Pontimonas | OTU6602 | | 0.38 |
| Planctomycetota | Rhodopirellula | OTU981 | 0.21 | Cyanobacteria | | Synechococcus_CC9902 | OTU16157 | | 0.37 |
|  |  | Total | 79.68 | Proteobacteria | | norank_f__Legionellaceae | OTU23079 | | 0.37 |
|  |  |  |  | Proteobacteria | | Labrenzia | OTU15612 | | 0.32 |
|  |  |  |  | Firmicutes | | ZOR0006 | OTU16551 | | 0.28 |
|  |  |  |  | Chloroflexi | | norank_f__norank_o__norank_c__KD4-96 | OTU2927 | | 0.27 |
|  |  |  |  | Proteobacteria | | Pseudomonas | OTU24521 | | 0.27 |
|  |  |  |  | Cyanobacteria | | Synechococcus_CC9902 | OTU18271 | | 0.27 |
|  |  |  |  | Firmicutes | | Bacillus | OTU21919 | | 0.26 |
|  |  |  |  | Cyanobacteria | | unclassified_f__Cyanobiaceae | OTU17841 | | 0.26 |
|  |  |  |  | Firmicutes | | Exiguobacterium | OTU18180 | | 0.25 |
|  |  |  |  | Firmicutes | | Staphylococcus | OTU8952 | | 0.24 |
|  |  |  |  | Planctomycetota | | norank_f__Pirellulaceae | OTU11937 | | 0.24 |
|  |  |  |  | Actinobacteriota | | norank_f__Nitriliruptoraceae | OTU5302 | | 0.24 |
|  |  |  |  | Proteobacteria | | Vibrio | OTU4771 | | 0.58 |
|  |  |  |  | Proteobacteria | | Pseudomonas | OTU18020 | | 0.23 |
|  |  |  |  | Planctomycetota | | Planctomicrobium | OTU16487 | | 0.22 |
|  |  |  |  | Proteobacteria | | Roseovarius | OTU16476 | | 0.11 |
|  |  |  |  | Planctomycetota | | norank_f__Pirellulaceae | OTU8993 | | 0.21 |
|  |  |  |  |  | |  | Total | | 78.99 |
| C (47 ± 0.62 | | | | D (55 ± 1.74) | | | | | |
| Phylum | Genus | OTU ID | mean (%) | | Phylum | Genus | OTU ID | mean (%) | |
| Cyanobacteria | Arthrospira_PCC-7345 | OTU10927 | 32.32 | | Cyanobacteria | Synechococcus_CC9902 | OTU1044 | 29.11 | |
| Cyanobacteria | Synechococcus_CC9902 | OTU1044 | 12.18 | | Cyanobacteria | Arthrospira_PCC-7345 | OTU10927 | 5.49 | |
| Firmicutes | norank_f__Mycoplasmataceae | OTU15284 | 4.46 | | Firmicutes | norank_f__Mycoplasmataceae | OTU15290 | 4.61 | |
| Planctomycetota | norank_f__Pirellulaceae | OTU5399 | 4.32 | | Planctomycetota | norank_f__Pirellulaceae | OTU5399 | 3.42 | |
| Planctomycetota | norank_f__Pirellulaceae | OTU1056 | 4.03 | | Actinobacteriota | DS001 | OTU5318 | 3.16 | |
| Planctomycetota | Blastopirellula | OTU11967 | 2.26 | | Cyanobacteria | norank_f__norank_o__Chloroplast | OTU25168 | 2.99 | |
| Firmicutes | Candidatus_Bacilloplasma | OTU360 | 1.80 | Proteobacteria | | Ralstonia | OTU1370 | 2.86 | |
| Proteobacteria | unclassified_c__Gammaproteobacteria | OTU8977 | 1.60 | Proteobacteria | | norank_f__Mitochondria | OTU11990 | 2.11 | |
| Firmicutes | Staphylococcus | OTU8952 | 1.53 | Cyanobacteria | | norank_f__norank_o__Chloroplast | OTU1151 | 1.90 | |
| Firmicutes | ZOR0006 | OTU16551 | 1.46 | Actinobacteriota | | norank_f__norank_o__PeM15 | OTU5407 | 1.76 | |
| Actinobacteriota | DS001 | OTU5318 | 1.39 | Planctomycetota | | norank_f__Pirellulaceae | OTU1056 | 1.62 | |
| Bacteroidota | Spongiimonas | OTU8966 | 1.35 | Planctomycetota | | Blastopirellula | OTU11967 | 1.37 | |
| Proteobacteria | Ralstonia | OTU1370 | 1.34 | Planctomycetota | | Rubinisphaera | OTU26758 | 1.18 | |
| Cyanobacteria | norank_f__norank_o__Chloroplast | OTU1151 | 1.26 | Firmicutes | | norank_f__Mycoplasmataceae | OTU15284 | 1.16 | |
| Firmicutes | Candidatus_Bacilloplasma | OTU75 | 1.20 | Planctomycetota | | Rhodopirellula | OTU981 | 1.14 | |
| Proteobacteria | unclassified_f__Rhodobacteraceae | OTU26347 | 1.10 | Proteobacteria | | Delftia | OTU24481 | 1.01 | |
| Actinobacteriota | norank_f__norank_o__PeM15 | OTU5407 | 0.93 | Proteobacteria | | Acinetobacter | OTU12281 | 0.97 | |
| Proteobacteria | Pseudomonas | OTU18484 | 0.74 | Bacteroidota | | norank_f__Balneolaceae | OTU1049 | 0.70 | |
| Planctomycetota | norank_f__Pirellulaceae | OTU11937 | 0.56 | Planctomycetota | | norank_f__Pirellulaceae | OTU8993 | 0.63 | |
| Planctomycetota | Rhodopirellula | OTU981 | 0.53 | Planctomycetota | | norank_f__Pirellulaceae | OTU11937 | 0.63 | |
| Proteobacteria | Legionella | OTU4426 | 0.48 | Proteobacteria | | norank_f__Legionellaceae | OTU23079 | 0.62 | |
| Planctomycetota | norank_f__Pirellulaceae | OTU8993 | 0.48 | Proteobacteria | | norank_f__Mitochondria | OTU1369 | 0.59 | |
| Verrucomicrobiota | LD29 | OTU1207 | 0.48 | Proteobacteria | | norank_f__norank_o__norank_c__KD4-96 | OTU3477 | 0.58 | |
| Firmicutes | Candidatus_Bacilloplasma | OTU18492 | 0.46 | Actinobacteriota | | ML602J-51 | OTU5336 | 0.49 | |
| Cyanobacteria | norank_f__norank_o__Chloroplast | OTU1362 | 0.45 | Proteobacteria | | unclassified_f__Rhodobacteraceae | OTU9090 | 0.49 | |
| Proteobacteria | norank_f__Mitochondria | OTU1369 | 0.41 | Proteobacteria | | Pseudomonas | OTU18484 | 0.49 | |
| Cyanobacteria | norank_f__norank_o__Chloroplast | OTU25168 | 0.40 | Planctomycetota | | Rhodopirellula | OTU17685 | 0.48 | |
| Cyanobacteria | Synechocystis_PCC-6803 | OTU5340 | 0.33 | Actinobacteriota | | Rhodococcus | OTU1385 | 0.48 | |
| Actinobacteriota | ML602J-51 | OTU5336 | 0.29 | Actinobacteriota | | norank_f__norank_o__Microtrichales | OTU15286 | 0.44 | |
| Proteobacteria | Ruegeria | OTU18270 | 0.29 | Proteobacteria | | Roseovarius | OTU11803 | 0.42 | |
| Proteobacteria | Tropicimonas | OTU8921 | 0.28 | Proteobacteria | | unclassified_f__Oxalobacteraceae | OTU3954 | 0.41 | |
| Cyanobacteria | Arthrospira_PCC-7345 | OTU24681 | 0.28 | Firmicutes | | norank_f__Mycoplasmataceae | OTU12107 | 0.39 | |
| Proteobacteria | unclassified_f__Rhodobacteraceae | OTU9090 | 0.28 | Proteobacteria | | unclassified_f__Rhodobacteraceae | OTU26347 | 0.38 | |
| Proteobacteria | Delftia | OTU24481 | 0.27 | Proteobacteria | | Xanthomonas | OTU12815 | 0.38 | |
| Proteobacteria | Roseovarius | OTU11803 | 0.27 | Cyanobacteria | | Synechocystis_PCC-6803 | OTU5340 | 0.37 | |
| Bacteroidota | norank_f__Flavobacteriaceae | OTU16646 | 0.24 | Firmicutes | | Bacillus | OTU10538 | 0.36 | |
| Verrucomicrobiota | Terrimicrobium | OTU5332 | 0.23 | Dependentiae | | unclassified_o__Babeliales | OTU12020 | 0.34 | |
| Desulfobacterota | Desulfonema | OTU23321 | 0.23 | Actinobacteriota | | norank_f__norank_o__PeM15 | OTU15328 | 0.29 | |
| Cyanobacteria | Synechococcus_CC9902 | OTU16157 | 0.23 | Actinobacteriota | | norank_f__norank_o__PeM15 | OTU18328 | 0.29 | |
| Proteobacteria | Vibrio | OTU1121 | 0.44 | Proteobacteria | | Acinetobacter | OTU17973 | 0.28 | |
| Actinobacteriota | Corynebacterium | OTU8978 | 0.20 | Cyanobacteria | | norank_f__norank_o__Chloroplast | OTU1362 | 0.28 | |
| Proteobacteria | Escherichia-Shigella | OTU16450 | 0.20 | Cyanobacteria | | Synechococcus_CC9902 | OTU16157 | 0.27 | |
|  |  | Total | 83.59 | Bacteroidota | | norank_f__Saprospiraceae | OTU5404 | 0.25 | |
|  |  |  |  | Proteobacteria | | Aeromonas | OTU3599 | 0.23 | |
|  |  |  |  | Actinobacteriota | | norank_f__Nitriliruptoraceae | OTU5362 | 0.23 | |
|  |  |  |  | Planctomycetota | | CL500-3 | OTU1976 | 0.23 | |
|  |  |  |  | Proteobacteria | | unclassified_c__Gammaproteobacteria | OTU8977 | 0.22 | |
|  |  |  |  | Proteobacteria | | Alphaproteobacteria_Incertae_Sedis | OTU8967 | 0.22 | |
|  |  |  |  | Proteobacteria | | Alphaproteobacteria_Incertae_Sedis | OTU3766 | 0.21 | |
|  |  |  |  | Verrucomicrobiota | | Terrimicrobium | OTU5332 | 0.20 | |
|  |  |  |  |  | |  | Total | 78.70 | |
